# Revealing subject-specific temporal patterns from longitudinal data

**DOI:** 10.1093/bioinformatics/btag658

**Published:** 2026-09-04

**Authors:** Christos Chatzis, David Horner, Rasmus Bro, Ann-Marie Malby Schoos, Morten A Rasmussen, Evrim Acar

**Affiliations:** Department of Data Science and Knowledge Discovery, Simula Metropolitan Center for Digital Engineering, Oslo, 0170, Norway; Faculty of Technology, Art and Design, Oslo Metropolitan University, Oslo, 0166, Norway; COPSAC, Copenhagen Prospective Studies on Asthma in Childhood, Herlev and Gentofte Hospital, University of Copenhagen, Copenhagen, 2820, Denmark; Department of Food Science, University of Copenhagen, Copenhagen, 1958, Denmark; COPSAC, Copenhagen Prospective Studies on Asthma in Childhood, Herlev and Gentofte Hospital, University of Copenhagen, Copenhagen, 2820, Denmark; Department of Pediatrics, Copenhagen University Hospital—Næstved, Slagelse and Ringsted, Slagelse, 4200, Denmark; Department of Pediatrics, Copenhagen University Hospital—Amager and Hvidovre, Hvidovre, 2650, Denmark; Department of Clinical Medicine, University of Copenhagen, Copenhagen, 2200, Denmark; COPSAC, Copenhagen Prospective Studies on Asthma in Childhood, Herlev and Gentofte Hospital, University of Copenhagen, Copenhagen, 2820, Denmark; Department of Food Science, University of Copenhagen, Copenhagen, 1958, Denmark; Department of Data Science and Knowledge Discovery, Simula Metropolitan Center for Digital Engineering, Oslo, 0170, Norway

## Abstract

**Motivation:**

Temporal multivariate data are ubiquitous in many domains, for instance, being collected over time at planned visits (every few months/years) in longitudinal cohorts, or every few minutes/hours in challenge tests. The analysis of such data often focuses on revealing the underlying temporal patterns common across subjects. However, there are subject-specific differences in temporal patterns, which hold the promise to enhance our understanding of underlying mechanisms and facilitate personalized approaches. Nevertheless, extracting subject-specific temporal patterns from longitudinal multivariate data reliably is an open challenge.

**Results:**

We introduce coupled matrix factorizations (CMFs) as effective tools to capture subject-specific temporal patterns focusing on two novel applications: analysis of longitudinal metabolomics data and sensitization data. Our analysis shows that CMF models reliably capture subject-specific (shape) differences in temporal patterns with the promise to reveal further insights compared to the state of the art. In metabolomics, CMF models reveal differences in metabolic responses of individuals (in a postprandial meal challenge) according to anthropometric and insulin sensitivity measures. In sensitization data analysis, CMF-based methods capture differences in temporal trajectories of children according to delivery/birth mode and atopic disease diagnosis. We demonstrate the reliability of extracted patterns using reproducibility and replicability.

**Availability and implementation:**

The code is available on github.com/cchatzis/Revealing-Subject-specific-Temporal-Patterns-from-Longitudinal-Data and doi.org/10.5281/zenodo.22084338. Clinical data are not publicly available due to privacy reasons. Data can be made available under a joint research collaboration by contacting COPSAC (administration@dbac.dk).

## 1 Introduction

Longitudinal multidimensional datasets are collected in many fields with the goal of extracting insights, capturing early risk markers of diseases, or understanding various conditions, e.g. exposures in early life. For instance, blood metabolites are measured over time during meal challenge tests to understand differences in metabolic responses of individuals and how those are related to cardiometabolic diseases (Berry *et al.* 2020); gut microbiome data collected over time are analysed to understand how groups of microbes change in time and links with various conditions, e.g. delivery/birth mode (Martino *et al.* 2021), dietary interventions (Ma and Li 2023). Similarly, sensitization to allergens during childhood has been studied to investigate associations with atopic diseases (Schoos *et al.* 2017, Thorsen *et al.* 2025). Despite differences in data characteristics, a common goal in many domains is to capture individual-specific temporal trajectories of the underlying patterns and to understand the reasons for and implications of individual differences. For instance, sensitization to certain food allergens has been shown to increase in early childhood and then decrease (Schoos *et al.* 2017). The temporal trajectory, however, is not necessarily the same for everyone, and differences may reveal important insights. Therefore, a crucial question is how to reliably capture individual-specific temporal trajectories of the underlying patterns (see Fig. 1).

**Figure 1:**
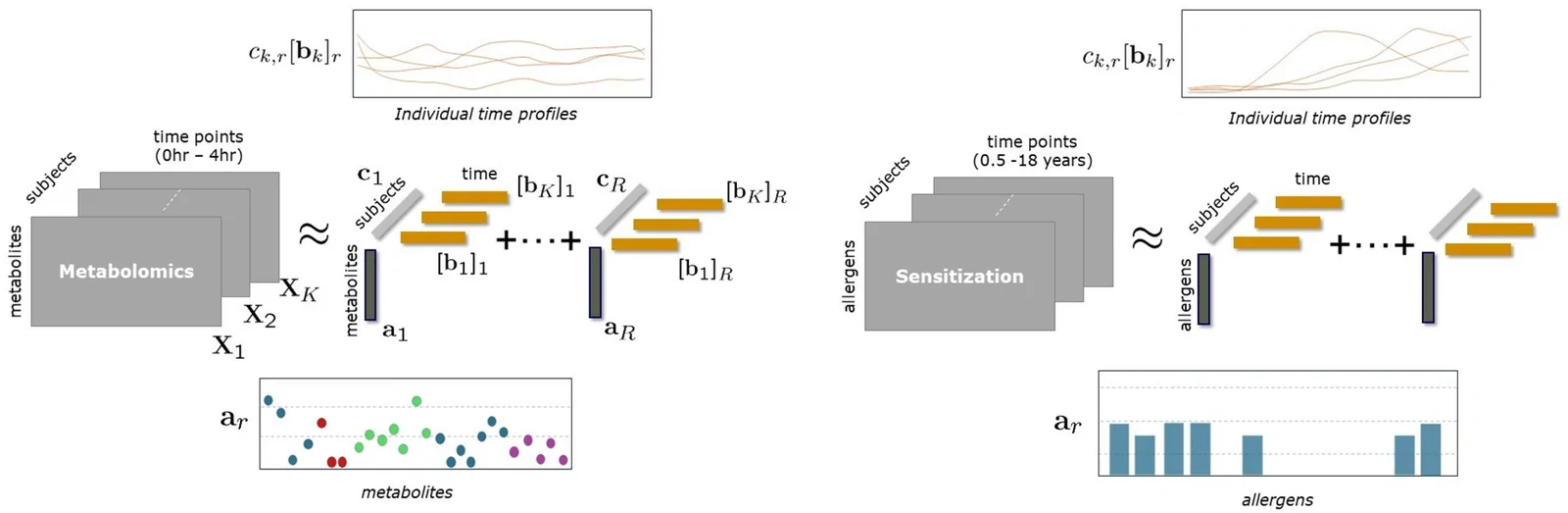
CMF models revealing subject-specific time profiles from a metabolites by time by subjects tensor, and an allergens by time by subjects tensor.

Multivariate measurements collected from multiple subjects over time form a multi-way array (in the case of aligned signals) or a collection of matrices. Multi-way arrays, also referred to as higher-order tensors, are generalizations of matrices to higher dimensions. For example, longitudinal measurements can be represented as a third-order tensor: features by time by subjects tensor as in Fig. 1. Traditional methods analysing such data focus on one time point at a time, one feature at a time or summary statistics (Metwally *et al.* 2022, Siroux *et al.* 2022). Recently, tensor decompositions have been used to analyse such longitudinal measurements. Tensor decompositions are extensions of matrix factorizations to higher-order tensors, and have been used to reveal the underlying patterns from such multi-way arrays in many fields with earlier work in chemometrics (Smilde *et al.* 2004) and later with widespread use in signal processing, neuroscience, and data science (Acar and Yener 2009, Ballard and Kolda 2025, Mørup *et al.* 2025). In longitudinal data analysis, in particular, the CANDECOMP/PARAFAC (CP) tensor model, which reveals reproducible (therefore, interpretable) patterns as a result of its uniqueness properties has been used. Here, uniqueness indicates that underlying patterns are the same up to scaling and permutation ambiguities when the model is fit to the data and the same approximation error is obtained. For instance, metabolomics data in the form of subjects by metabolites by time tensor have been analysed using a CP model extracting patterns in metabolite and time modes that reveal BMI (body mass index)-related differences (Yan *et al.* 2024). Similarly, underlying patterns in taxa and time mode extracted from longitudinal microbiome data using CP-based approaches have revealed differences, e.g. associated with delivery/birth mode (Martino *et al.* 2021, Ma and Li 2023, Shi *et al.* 2024, van der Ploeg *et al.* 2025). CP has also been used to reveal sensitization patterns, i.e. clusters of allergens, and their temporal trajectories from longitudinal sensitization data (Schoos *et al.* 2017, Thorsen *et al.* 2025). However, CP-based models extract common patterns across subjects and except for a scaling difference, they do not reveal the individual variability, e.g. shape differences, in those patterns.

In this article, our goal is to capture individual-specific temporal trajectories of the underlying patterns from longitudinal data. We demonstrate that coupled matrix factorizations (CMFs) are effective tools to capture such individual differences. We focus on two novel applications: analysis of longitudinal metabolomics data and sensitization data. In metabolomics, by accounting for subject variability in temporal profiles, CMF models reveal differences in the metabolic response of individuals according to BMI and IR (insulin resistance) measures. In sensitization data analysis, CMF models reveal differences in temporal trajectories of children according to birth mode, i.e. children born by C-section show earlier sensitization to a specific group of food allergens compared to those born by natural birth. We use CMF models with nonnegativity constraints to reveal reproducible patterns. We discuss the reliability of extracted patterns in terms of reproducibility and replicability (Adali *et al.* 2022, Mørup *et al.* 2025). Previously, PARAllel FACtor analysis 2 (PARAFAC2), which is a CMF-based approach with specific constraints and uniqueness properties, has been used to reveal subject-specific time profiles in other domains (Madsen *et al.* 2017, Perros *et al.* 2019, Mørup *et al.* 2025, Erdos *et al.* 2026). We assess the performance of CMF-based models including PARAFAC2 and discuss our findings in comparison with CP models.

## 2 Materials and methods

### 2.1 Datasets

We use two longitudinal datasets from the COPSAC2000 cohort, which consists of 411 subjects (with mothers with a history of asthma) (Bisgaard 2004). The first dataset contains metabolomics measurements of blood samples collected during a meal challenge test at the 18-year-old visit. The second dataset corresponds to allergen-specific immunoglobulin E (sIgE) measurements to allergens over the first 18 years.

#### 2.1.1 Metabolomics data

Two hundred and ninety-nine participants took part in the meal challenge test consuming a meal after overnight fasting. Blood samples were collected from participants at eight time points at 0 h (fasting state), 0.25 h, 0.5 h, 1 h, 1.5 h, 2 h, 2.5 h, and 4 h after meal intake. Plasma samples were measured using nuclear magnetic resonance spectroscopy providing a set of metabolites. We also included insulin and C-peptide hormone measurements. Details about the meal challenge test and data have previously been described (Yan *et al.* 2024).

Metabolomics data are arranged as a metabolites by time by subjects tensor (Fig. 1). We removed several subjects as outliers as in previous studies (Li *et al.* 2024, Yan *et al.* 2024). The data corresponding to males are the 161 metabolites by 8 time points by 140 males tensor. For females, there are two subjects with missing samples. A missing sample results in missing entries for all metabolites at a time point, which introduces a missing column in $X_{k}$ in Fig. 1. We removed those two subjects and analysed the 161 metabolites by 8 time points by 150 females tensor (see Section 4 for including subjects with missing samples). We analysed males and females separately due to previously observed sex-related differences in metabolic responses (Yan *et al.* 2024). Previous studies on these measurements focused on revealing subject stratifications based on fasting state data and the metabolic response in dynamic state; therefore, they analysed fasting state-corrected data (i.e. measurements at 0 h were subtracted from other time points) (Li *et al.* 2024, Yan *et al.* 2024). Here, we analysed the data without fasting state correction since we are interested in individual temporal trajectories. Before the analysis, the data were scaled within metabolites mode by dividing each metabolite slice by root mean square of that slice.

Additional data on subjects including weight, height, BMI, IR, body composition measures are available. We use these measures to study the relation between extracted patterns and phenotypes of interest (e.g. BMI-related) and to demonstrate differences in temporal trajectories of various groups, e.g. no IR, higher BMI versus IR, higher BMI. Since there are more patterns related to BMI-related phenotypes in males than females, our experiments focus on the analysis of data from males. We discuss the results from females briefly.

Meal challenge tests generating longitudinal metabolomics data have been commonly used in nutrition and health research (Wopereis 2023). Individual-specific temporal trajectories of the underlying patterns extracted from the data can better characterize the difference between subject stratifications while also paving the way for personalized nutrition (Jansen *et al.* 2012, Wopereis 2023). That is what we aim to demonstrate through the metabolomics application.

#### 2.1.2 Sensitization data

Blood samples were collected at ages 0.5, 1.5, 4, 6, 13, and 18 years, and sIgE levels were measured against five food allergens (milk, egg, wheat flour, peanut, and soybean), eight aeroallergens (birch, timothy grass, mugworth, dog, cat, horse, mold, and house dust mite). Due to missing measurements for soybean and horse for everyone at the last time point, we omitted those two allergens in our analysis.

Sensitization data are arranged as a third-order tensor with modes: allergens, time points, and subjects (Fig. 1). Out of 411 children, 337 of them have a nonzero sIgE value. We included only subjects with complete information, i.e. with sIgE measurements at 6 time points for all 11 allergens. The data are in the form of a 11 allergens by 6 time points by 176 subjects tensor.

Sensitization is often defined as $\text{sIgE}\geq 0.35{\text{kU}}_{A}/L$ and previous studies treated the data as binary data indicating whether there was sensitization, i.e. sIgE ≥ 0.35 or not (Schoos *et al.* 2017, Thorsen *et al.* 2025). Rather than using a cutoff value which may have limitations (Schoos *et al.* 2020), we used the measured sIgE values ranging between 0 and 384.6 ${\text{kU}}_{A}/L$. The data were then preprocessed by log-transform, i.e. $x_{\mathrm{ijk}}=\text{log}(x_{\mathrm{ijk}}+1)$ for the analysis not to be dominated by large values, where $x_{\mathrm{ijk}}$ is the (i,j,k)th entry of allergens by time by subjects tensor X containing sIgE values. Before the analysis, X was also scaled within allergens mode.

Previously, the sensitization data were analysed using a CP model to characterize the underlying sensitization patterns, i.e. allergen groups and their temporal profiles, and to relate them to atopic diseases (Schoos *et al.* 2017, Thorsen *et al.* 2025). These studies revealed aeroallergen and food allergen-dominant patterns and demonstrated how those patterns peaked at different ages or continued to increase over time. Those temporal profiles characterize the overall behavior in the cohort, while individual time trajectories showing whether/when individuals grow out of allergies may be quite different. Through the analysis of sensitization data, we aim to capture such individual temporal trajectories and understand potential conditions that may lead to differences.

### 2.2 Coupled factorizations

Let $X_{k}\in R^{I\times J}$ correspond to longitudinal multivariate measurements from subject *k*, i.e. *I* features measured at *J* time points. For *K* subjects, matrices $X_{k}$, k=1,…,K, can be jointly analysed to extract the underlying patterns using coupled factorizations. Coupled factorizations are effective approaches for capturing interpretable patterns from multi-way, multi-modal, multi-set data. They accommodate a variety of modeling assumptions, including different coupling relations (Mørup *et al.* 2025). Here, we focus on CP, PARAFAC2, and CMF models, which fall under coupled factorizations, and differ in terms of coupled modes and the coupling relation between $X_{k}$ matrices.

#### 2.2.1 CANDECOMP/PARAFAC

Matrices ${\left\{X_{k}\right\}}_{k=1,\ldots ,K}$ of size I×J can be arranged as a third-order tensor $X\in R^{I\times J\times K}$. Tensor factorizations have been successfully used to extract the underlying patterns from such multi-way data in many disciplines (Smilde *et al.* 2004, Acar and Yener 2009, Ballard and Kolda 2025). One of the most commonly used tensor models is the CP model (Carroll and Chang 1970, Harshman 1970) as a result of its uniqueness properties (Kruskal 1977). Uniqueness enables to reveal reproducible patterns, which can then be interpreted to extract further insights. An *R*-component CP model approximates the data as follows:


(1) X≈∑r=1Rar o br o cr=〚A,B,C〛,

where $A\in {\mathbb{R}}^{I\times R}=[a_{1}\;a_{2}\;\ldots \;a_{R}]$, $B\in {\mathbb{R}}^{J\times R}=[b_{1}\;b_{2}\;\ldots \;b_{R}]$, and $C\in {\mathbb{R}}^{K\times R}=[c_{1}\;c_{2}\;\ldots \;c_{R}]$ correspond to factor matrices in each mode; o denotes the outer product. CP is unique up to permutation and scaling ambiguities under mild conditions, which means that *R* components can be permuted, and within each component, i.e. ar o br o cr, the vectors in each mode can be scaled as long as the product of their norms stay the same (Ballard and Kolda 2025). These ambiguities do not interfere with the interpretation.

In slice-wise matrix notation, the CP model corresponds to $X_{k}\approx AD_{k}B^{T}$, where $D_{k}\in R^{R\times R}$ is a diagonal matrix with *k*th row of C on the diagonal. If $X_{k}$ corresponds to allergens by time points sensitization data for the *k*th subject, $a_{r}$ captures the *r*th allergen pattern, $b_{r}$ corresponds to its temporal profile while $c_{r}$ shows the subject scores indicating how much *r*th pattern shows up in each subject. The CP model assumes that $X_{k}$ matrices are coupled in both allergens and time modes, and extracts the same factor matrices A and B for all subjects, k=1,…,K.

In our experiments, we fit CP models with nonnegativity constraints in all modes by solving the following problem:


(2) $$\begin{array}{c} \underset{A,B,{\left\{D_{k}\right\}}_{k\leq K}}{\text{min}}\sum_{k=1}^{K}{\left\|X_{k}-AD_{k}B^{T}\right\|}_{F}^{2},\\ \;\;\;\;\;s.t.\;A,B,{\left\{D_{k}\right\}}_{k\leq K}\geq 0 \end{array}$$

#### 2.2.2 PARAFAC2

PARAFAC2 (Harshman 1972) relaxes the coupling assumption in the CP model, and jointly analyses matrices ${\left\{X_{k}\right\}}_{k=1,\ldots ,K}$ using an *R*-component model as follows:


(3) $$\begin{array}{cc} X_{k} & \approx AD_{k}B_{k}^{T},\;\;\;B_{k}\in P,\;\;\;\text{for}\;\;k=1,\ldots ,K,\\ & P=\left\{{\left\{B_{k}\right\}}_{k=1}^{K}|B_{k}^{T}B_{k}=\Phi ,\;k=1,\ldots ,K\right\}, \end{array}$$

 where each $X_{k}$ is modeled using its own $B_{k}\in R^{J\times R}$ matrix, rather than the same B for all $X_{k}$s (Fig. 1). Together with the cross-product constraint on $B_{k}$ matrices in (3), also referred to as the PARAFAC2 constraint, where $\Phi \in R^{R\times R}$ is the same for all *k*, the model is unique up to permutation and scaling ambiguities (Kiers *et al.* 1999). For an allergens by time points sensitization matrix $X_{k}$ for the *k*th subject, $a_{r}$ captures the *r*th allergen pattern, ${\left[b_{k}\right]}_{r}$ corresponds to the temporal profile of that pattern for subject *k*, where ${\left[b_{k}\right]}_{r}$ denotes the *r*th column of $B_{k}$, and $c_{r}$ shows the subject scores for pattern *r*. As a result of its uniqueness properties which enable reproducible pattern discovery and facilitate interpretation, PARAFAC2 has been previously used in many applications. In chemometrics, it was used to separate mixtures measured using gas chromatography–mass spectrometry by accounting for differences in elution profiles of chemicals across mixtures (Amigo *et al.* 2008). PARAFAC2 was also used to reveal subject-specific temporal patterns in neuroimaging data analysis (Madsen *et al.* 2017, Mørup *et al.* 2025), to extract patient-specific temporal trajectories from EHR (electronic health records) (Perros *et al.* 2019) and to capture subject-specific temporal patterns in longitudinal microbiome data analysis (Erdos *et al.* 2026). The additional expressivity of PARAFAC2 over CP in capturing subject-specific temporal patterns has been demonstrated on both synthetic and real data (Erdos *et al.* 2026).

In this article, we use PARAFAC2 models with nonnegativity constraints in all modes and solve the following problem:


(4) $$\begin{array}{c} \underset{A,{\left\{D_{k},B_{k}\right\}}_{k\leq K}}{\text{min}}\sum_{k=1}^{K}{\left\|X_{k}-AD_{k}B_{k}^{T}\right\|}_{F}^{2}\\ \;\;\;\;\;s.t.\;A,{\left\{B_{k},D_{k}\right\}}_{k\leq K}\geq 0,{\left\{B_{k}\right\}}_{k\leq K}\in P \end{array}$$

#### 2.2.3 Coupled matrix factorizations

Given matrices ${\left\{X_{k}\right\}}_{k=1,\ldots ,K}$ of size I×J coupled in one mode, e.g. the first mode, they can be jointly analysed using CMFs (also known as collective matrix factorizations (Singh and Gordon 2008)) using *R* components as follows:


(5) $$X_{k}\approx AB_{k}^{T}$$

This formulation cannot uniquely recover factor matrices $A\in R^{I\times R}$ and ${\left\{B_{k}\right\}}_{k\leq K}\in R^{J\times R}$. However, CMF models with additional constraints have been used in various applications to find the underlying patterns, e.g. with nonnegativity constraints on all factor matrices to analyse multiple gene expression datasets (Badea 2008), with nonnegativity and sparsity constraints on A, temporal smoothness on ${\left\{B_{k}\right\}}_{k\leq K}$ to extract patient-specific temporal profiles in EHR analysis (Zhou *et al.* 2014). In our analysis, we use CMF models with nonnegativity constraints in all modes by solving the following problem:


(6) $$\begin{array}{c} \underset{A,{\left\{B_{k}\right\}}_{k\leq K}}{\text{min}}\sum_{k=1}^{K}{\left\|X_{k}-AB_{k}^{T}\right\|}_{F}^{2}\\ \;\;\;\;\;s.t.\;A,{\left\{B_{k}\right\}}_{k\leq K}\geq 0 \end{array}$$

This is a coupled nonnegative matrix factorization problem which, as we see in our reproducibility results, achieves unique results as a result of nonnegativity constraints and coupling. For $X_{k}$ corresponding to allergens by time points sensitization data for the *k*th subject, $a_{r}$ captures the *r*th allergen pattern while ${\left[b_{k}\right]}_{r}$ corresponds to the temporal profile of that pattern for subject *k* (as in PARAFAC2) (Fig. 1). Subject scores for pattern *r* can be obtained by setting $c_{k,r}=\left\|a_{r}\right\|\left\|{\left[b_{k}\right]}_{r}\right\|$ and then normalizing $c_{r}$. This model is less constrained than (4) since the cross-product constraint $B_{k}^{T}B_{k}=\Phi$ is dropped not to distort subject-specific temporal profiles due to the constraint.

Nonnegativity constraints can be imposed in all models here because datasets of interest in this article, i.e. metabolomics and sensitization data, are nonnegative. In addition to improving uniqueness properties, nonnegativity constraints offer benefits in terms of interpretation, e.g. components become additive with no cancelation effects; nonnegative quantities such as subject participation and temporal profiles remain nonnegative in the latent space.

### 2.3 Experimental set-up

We analyse metabolomics and sensitization data using CP, PARAFAC2, and CMF models by solving the optimization problems in (2), (4), and (6). We fit the models using different number of components (*R*), and assess their reproducibility and replicability—which is crucial for discovering patterns and to be able to interpret them (Adali *et al.* 2022, Mørup *et al.* 2025). Reproducibility refers to the ability to uncover a unique set of patterns/components given the same data. We investigate this ability of each model by comparing the solutions obtained by different initializations reaching the same (minimum) objective function value. Replicability refers to the ability of a model to consistently recover similar patterns across different datasets. In this article, we assess replicability by comparing the patterns extracted from different subsets of the data.

The similarity between two solutions is quantified using factor match scores (FMSs):


(7) $$\mathrm{FMS}A=\frac{1}{R}\sum_{r=1}^{R}\frac{\left|a_{r}^{\left(1\right)}{\;}^{⊤}a_{r}^{\left(2\right)}\right|}{\left\|a_{r}^{\left(1\right)}\|\|a_{r}^{\left(2\right)}\right\|}$$

(8) $$\mathrm{FMS}\mathrm{C*B}=\frac{1}{R}\sum_{r=1}^{R}\frac{\left|v_{r}^{\left(1\right)}{\;}^{⊤}v_{r}^{\left(2\right)}\right|}{\left\|v_{r}^{\left(1\right)}\|\|v_{r}^{\left(2\right)}\right\|}\;\text{with}$$

$$\begin{array}{c} v_{r}^{\left(1\right)}=\left[\begin{array}{c} c_{1,r}^{\left(1\right)}{\left[b_{1}^{\left(1\right)}\right]}_{r}\\ \vdots \\ c_{k,r}^{\left(1\right)}{\left[b_{k}^{\left(1\right)}\right]}_{r}\\ \vdots \\ c_{K,r}^{\left(1\right)}{\left[b_{K}^{\left(1\right)}\right]}_{r} \end{array}\right]\in R^{KJ}\;\text{and}\;v_{r}^{\left(2\right)}=\left[\begin{array}{c} c_{1,r}^{\left(2\right)}{\left[b_{1}^{\left(2\right)}\right]}_{r}\\ \vdots \\ c_{k,r}^{\left(2\right)}{\left[b_{k}^{\left(2\right)}\right]}_{r}\\ \vdots \\ c_{K,r}^{\left(2\right)}{\left[b_{K}^{\left(2\right)}\right]}_{r} \end{array}\right]\in R^{KJ}\;, \end{array}$$

where superscripts (1) and (2) denote different factorizations (i.e. solutions), and $c_{k,r}{\left[b_{k}\right]}_{r}$ denotes the scaled time profiles for the *k*th subject for the *r*th component. FMS_A_ captures the (absolute) cosine similarity between the first mode factor matrices (e.g. metabolites or allergens), FMS_C*B_ quantifies the similarity between the scaled time profiles. Both metrics are computed after matching the order of components. An FMS value of 1.0 indicates perfect match between two models.

When assessing reproducibility, we compare the solutions from different models in terms of FMS_A_ and FMS_C*B_. For replicability, we use the following procedure:

1. Randomly split the data in 10 (possibly stratified) folds.
2. Form 10 subsets of the data by removing each fold from the complete dataset.
3. Fit the model to each subset.
4. Compare the models (in terms of FMS_A_ and FMS_C*B_) across different subsets.
5. Repeat steps (1–4) 10 times.

From this procedure, $450\;(=10\times \left(\begin{array}{c} 10\\ 2 \end{array}\right))$ FMS_A_ and 450 FMS_C*B_ values are computed. When comparing models from different subsets of data, only the common subjects are used in FMS_C*B_ computations (Erdos *et al.* 2026). We consider that models are replicable when 95% of the FMS values are above 0.90.

#### 2.3.1 Implementation details

For the CP model, we use non_negative_parafac function from TensorLy (Kossaifi *et al.* 2019) using block coordinate multiplicative updates. For CMF and PARAFAC2, we use MatCoupLy (Roald 2023), which utilizes AO-ADMM (alternating optimization—alternating direction method of multipliers) (Roald *et al.* 2022) with Expectation Maximization to handle missing data (Chatzis *et al.* 2025b) (missing data is only present in metabolomics, i.e. 0.07% of the entries in males and 0.08% in females). We use MatCoupLy since the AO-ADMM framework in MatCoupLy enables to impose nonnegativity constraints on all modes. For each experiment, 50 random initializations were used, and the best run was chosen as the one reaching the minimum function value after discarding runs that reached the maximum number of iterations (set to 15 000). For AO-ADMM, all runs with feasibility gaps larger than $10^{-5}$ at termination were discarded. The algorithms stopped due to either small absolute ($10^{-6}$) or relative ($10^{-8}$) change in the total loss.

## 3 Results

### 3.1 Longitudinal metabolomics data analysis

Figure 2 shows the components extracted using a 6-component CMF model of tensor X by solving (6), where $X_{k}$s correspond to 161 metabolites × 8 time points matrices, and K=140 subjects (see Section 3.1.1 for the selection of *R*). For each component *r*, the metabolite pattern ($a_{r}$) and subject-specific time profiles scaled by subject scores ($c_{k,r}{\left[b_{k}\right]}_{r}$) are shown. Metabolites are colored according to lipoprotein classes, i.e. HDL (high density lipoprotein), IDL (intermediate density lipoprotein), LDL (low density lipoprotein), and VLDL (very low density lipoprotein). Glycolysis-related metabolites, ketone bodies, insulin, and c-peptide are also marked. The last column shows the scaled subject-specific time profiles colored according to groups considering both BMI and IR, where BMI and IR groups are defined as: lower BMI: BMI <25, higher BMI: BMI ≥25; NoIR: HOMA-IR ≤2.91, IR: HOMA-IR >2.91 (Silva *et al.* 2022).

**Figure 2:**
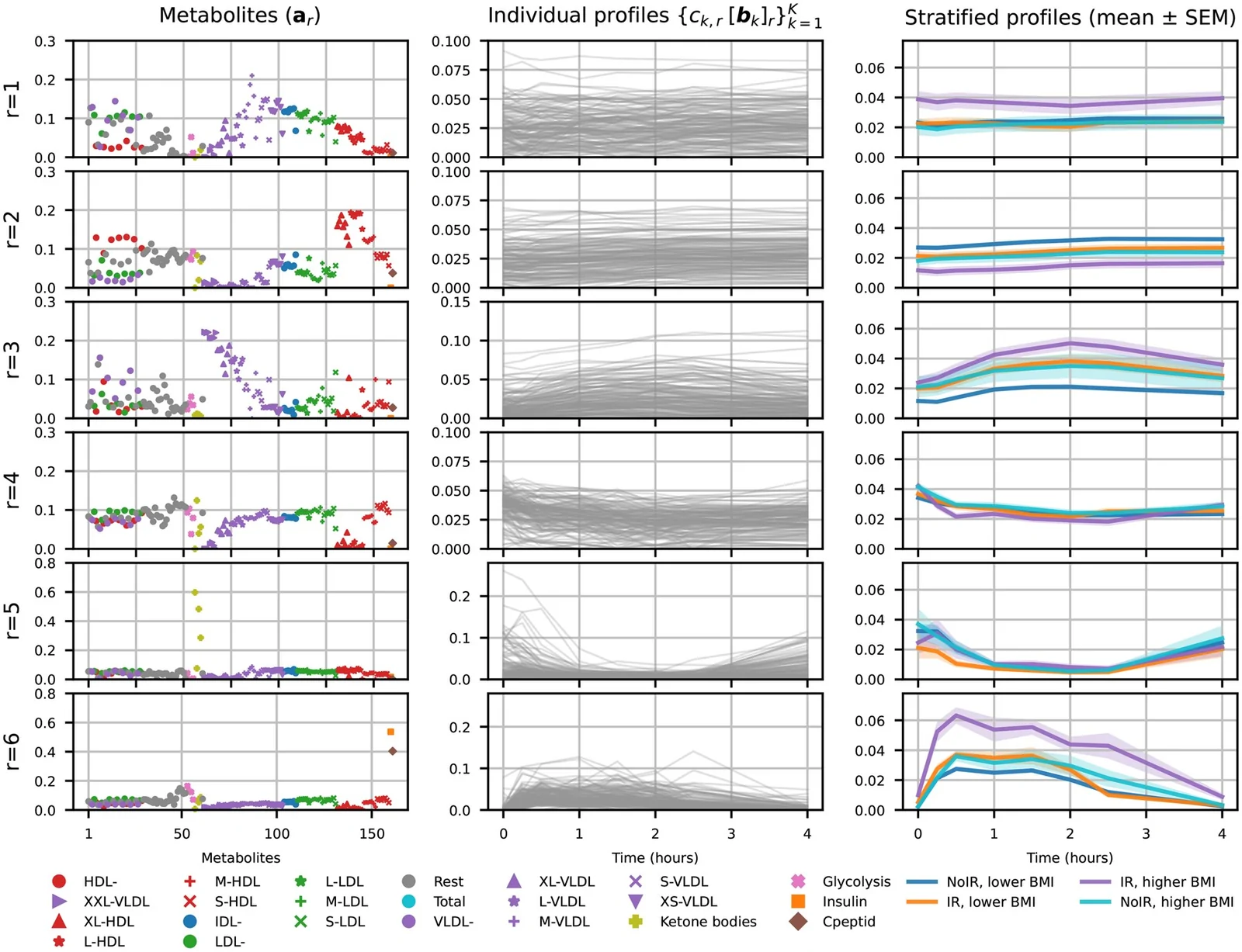
Metabolomics. Components of a 6-component CMF model (with nonnegativity constraints in all modes) of the metabolomics data from males. $a_{r}$ denotes the pattern in the metabolites mode, where metabolites are colored by lipoprotein classes. Different shapes are used for lipoprotein subclasses. Subject-specific time profiles scaled by the corresponding subject scores, i.e. $c_{k,r}{\left[b_{k}\right]}_{r}$, for each component are shown in the middle column. The last column shows scaled subject-specific time profiles colored according to four BMI/IR groups. On the *y*-axis, the label r=1 denotes component 1, r=2 denotes component 2, etc.

Out of six components, subject scores ($c_{r}$) of four components (components 1, 2, 3, and 6) show statistically significant difference in terms of BMI groups (Table S.3, available as supplementary data at *Bioinformatics* online). Component 1 mainly captures the response of smaller size (XS, S, M) VLDL, IDL, and LDL while component 3 models larger size (XXL, XL, and L) VLDL. Subject-specific time profiles show the difference in the response of each subject in terms of these metabolites. In the third column, we observe that both BMI and IR play a role in these differences, with component 1 showing a sustained elevation in the IR, higher-BMI group throughout the postprandial window, and component 3 showing a profile peaking around 2 h, most pronounced in the same group. Component 2 models HDL, and at the group level, we observe the reverse ordering of BMI/IR subgroups—with HDL being negatively associated with the (IR, higher BMI) group. Component 6 captures the response of insulin, c-peptide, and also picks up glycolysis-related metabolites (glucose, lactate, pyruvate) and some aminoacids (isoleucine, leucine). The second column shows the individual differences in the time profiles, and the last column shows how the response peaks and decreases for different groups. Among the four components related to BMI, component 6 achieves the highest correlation. Figure 3 shows the correlations between subject scores ($c_{6}$) and BMI and other variables of interest. We have not observed any association between these variables and components 4 and 5. In females, similar components as in Fig. 2 are revealed using a 6-component CMF model. Despite similar components (modeling lipoproteins), only the component modeling insulin, c-peptide, and glycolysis-related metabolites has good correlations with BMI (and other variables) supporting previously-reported sex-differences (Yan *et al.* 2024). Due to space limitations, we provide the results for females in Figs S.5–S.7, available as supplementary data at *Bioinformatics* online. We have also provided the analysis of males and females jointly in SM in Figs S.9–S.11, available as supplementary data at *Bioinformatics* online. With the exception of the insulin/c-peptide component, lipoprotein-dominated components show sex-dependent associations with BMI, making their relation to BMI difficult to characterize when sexes are pooled (Table S.5 versus Table S.4, available as supplementary data at *Bioinformatics* online). This confirms that analysing males and females separately better reveals the association between patterns of metabolic response to a meal challenge test and BMI.

**Figure 3:**
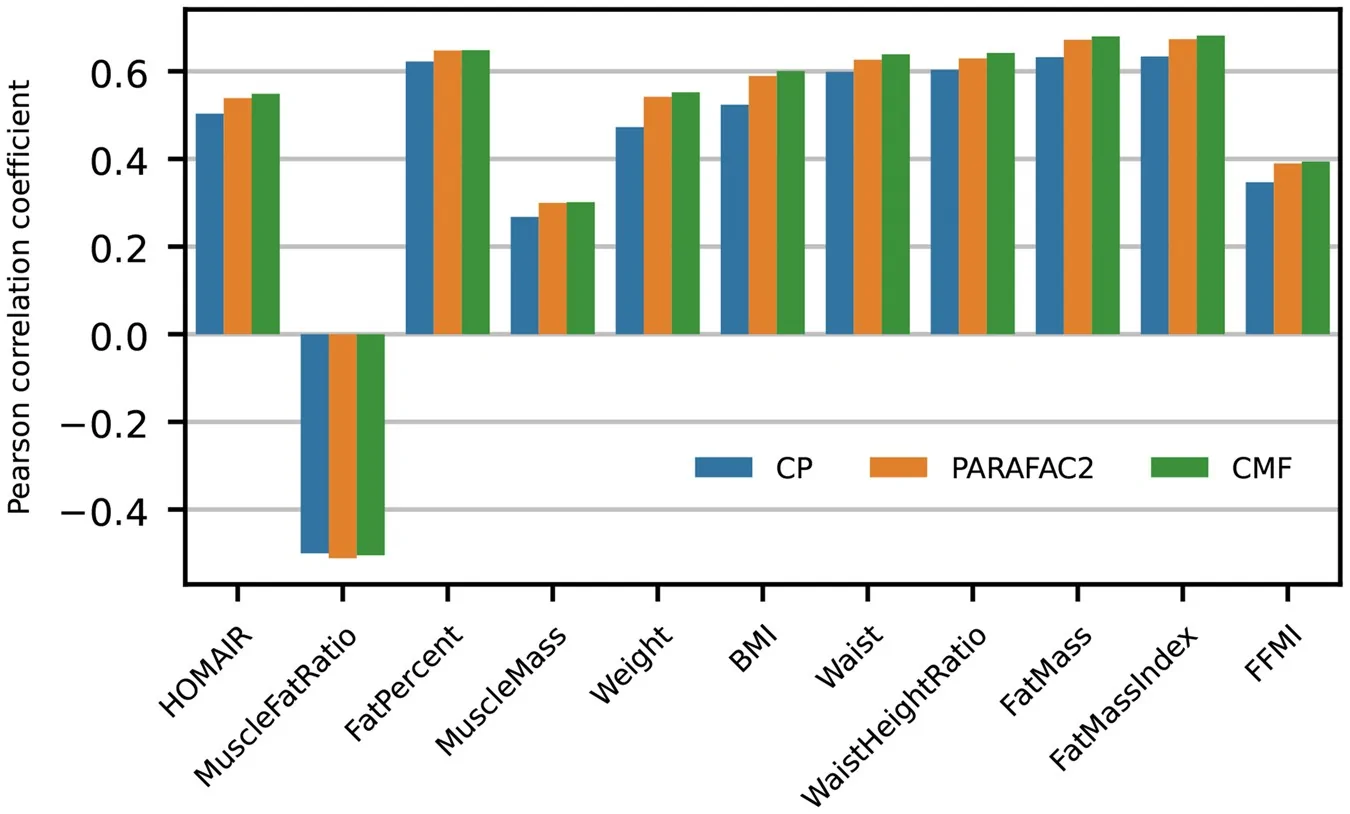
Correlation between variables of interest and (male) subject scores ($c_{r}$) for the component modeling insulin, c-peptide, and glycolysis-related metabolites. HOMA-IR, homeostatic model assessment for insulin resistance; MuscleFatRatio denotes muscle to fat ratio; FatPercent denotes body fat percentage; MuscleMass indicates the amount of muscle in the body; Waist denotes the waist circumference; WaistHeightRatio is the waist measurement divided by height; FatMass denotes the amount of body fat; FatMassIndex is computed as FatMass/height^2^; and FFMI indicates the fat free mass index. See Yan *et al*. (2024) for more details.

We compare the CMF model with a 6-component CP and a 6-component PARAFAC2 model of tensor X (see Section 3.1.1 for component number selection). In the metabolites mode, both CP and PARAFAC2 capture patterns similar to those captured by CMF. Figure 4 shows the component modeling insulin, c-peptide, and glycolysis-related metabolites captured by CP and PARAFAC2 (for all components, see Figs S.2 and S.3, available as supplementary data at *Bioinformatics* online). In terms of time profiles, CP extracts the same time profile for every subject; therefore, the response has the same shape for everyone and is only scaled by subject scores. The model fails to capture shape differences between individuals and between the BMI/IR groups. PARAFAC2, on the other hand, extracts subject-specific profiles and captures shape differences between BMI/IR groups (similar to component 6 in the CMF model). Those shape differences may reveal biologically relevant insights. A healthy insulin curve after a meal is normally biphasic, showing rapid insulin secretion followed by slower insulin release. This biphasic response is also observed for glucose and c-peptide. When the biphasic response turns into a monophasic response, this has been associated with IR (Kim *et al.* 2016). We may be observing these differences in the temporal patterns when we compare CP versus CMF/PARAFAC2. However, these findings should be considered hypothesis-generating and warrant further investigation in independent cohorts. Note that component 6 is not just modeling insulin but also c-peptide and glycolysis-related metabolites. Similarly, other components model groups of metabolites. Therefore, CMF/PARAFAC2 models reveal time profiles showing differences due to those groups of metabolites—not for one metabolite at a time.

**Figure 4:**
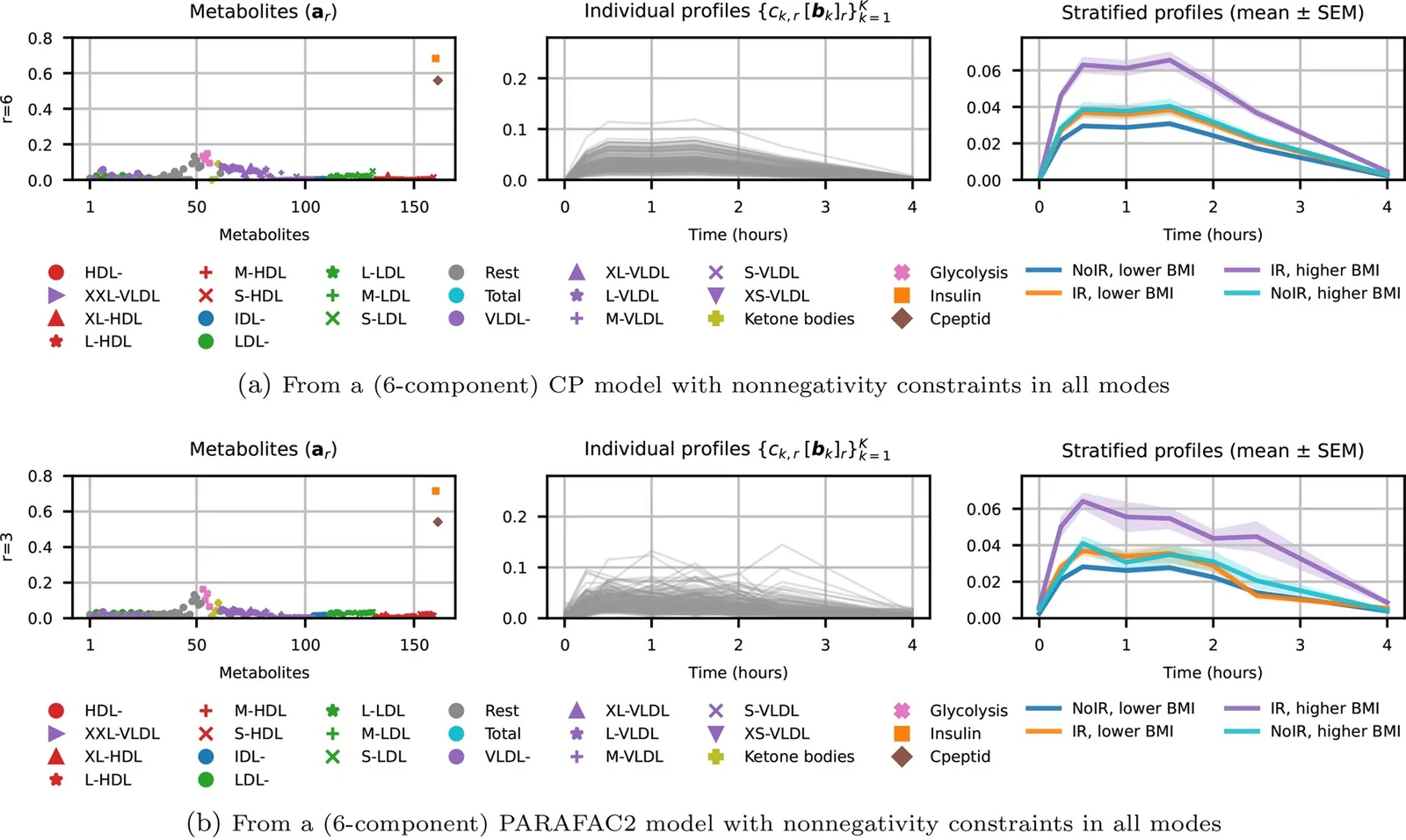
The component modeling insulin, c-peptide, and glycolysis-related metabolites extracted using different models. $a_{r}$ denotes the pattern in the metabolites mode. Subject-specific time profiles scaled by the corresponding subject scores, i.e. $c_{k,r}{\left[b_{k}\right]}_{r}$, are shown in the middle column. Scaled subject-specific time profiles colored according to BMI/IR groups are shown in the last column. Note that due to permutation ambiguity, components in different models may have different orders. Therefore, this component corresponds to component 6 (marked with r=6 on *y*-axis label) for CP while it is component 3 (r=3) in PARAFAC2.

Figure 3 shows the correlations between subject scores for the component modeling primarily insulin and c-peptide, and variables of interest. All methods achieve similar correlations indicating that subject scores from different models are similar and not enough to capture such differences in temporal trajectories. However, methods differ in terms of subject-specific time profiles (see Fig. S.4, available as supplementary data at *Bioinformatics* online, for the similarity between time profiles among subjects for each method). If we use the scaled subject-specific temporal profiles $c_{k,r}{\left[b_{k}\right]}_{r}$ as regressors to predict BMI and HOMA-IR (Tables S.1 and S.2, available as supplementary data at *Bioinformatics* online), the additional flexibility of CMF and PARAFAC2 improves prediction over CP, increasing $R^{2}$ for BMI from 0.23 (CP) to 0.39 (PARAFAC2) and 0.41 (CMF), and for HOMA-IR from 0.21 (CP) to 0.28 (PARAFAC2) and 0.32 (CMF). Such performance improvement is also observed for other BMI-related components.

Previous studies analysed these measurements using CP focusing on fasting state-corrected data (Yan *et al.* 2024), and its joint analysis with fasting data (Li *et al.* 2024). They revealed BMI-related components dominated by lipoproteins as in components 1, 2, and 3 in Fig. 2. However, the component modeling insulin, c-peptide, and glycolysis-related metabolites was not captured achieving lower correlations with variables of interest. In this article, we capture the response of these important metabolites, and also, via PARAFAC2 and CMF, the subject-specific time profiles.

#### 3.1.1 Reproducibility and replicability

Figure 5a shows the reproducibility of patterns extracted by CP, PARAFAC2, and CMF models using different *R*. With FMS_A_ and FMS_C*B_ close to 1, all models with 2–8 components produce unique results and are reproducible. Figure 5b shows the replicability of extracted patterns across different subsets of the data obtained via stratified sampling based on BMI groups. Except for PARAFAC2 with eight components, all models can be considered replicable. We choose R=6 for all models based on replicability and interpretation of components. Up to five components, insulin/c-peptide component cannot be captured. With R=6, a cleaner insulin/c-peptide component is captured, compared to R=5. However, for R=7, we see components splitting into multiple components.

**Figure 5:**
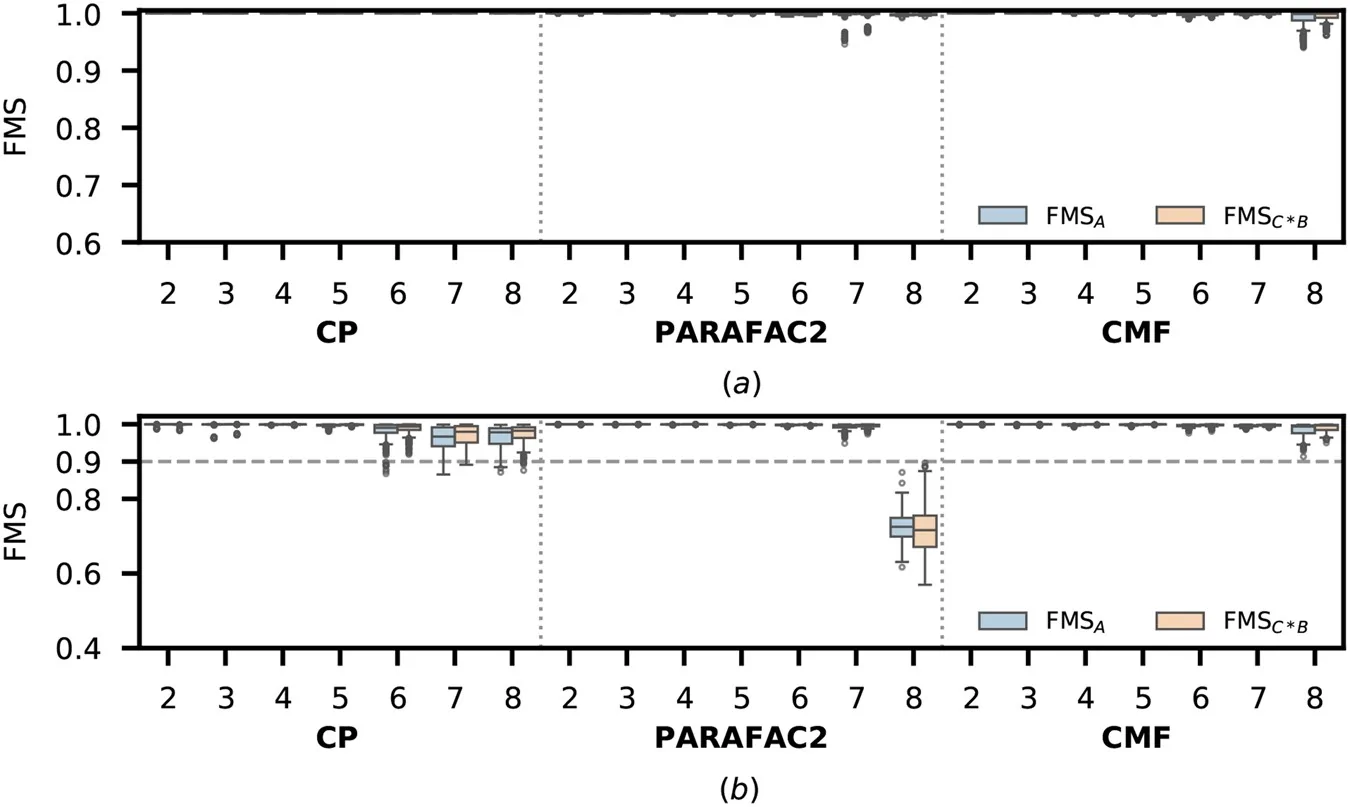
(a) Reproducibility and (b) replicability of different models of the metabolomics data using different *R*.

### 3.2 Longitudinal sensitization data analysis

Figure 6 shows the components of a 5-component CMF model of tensor X, where $X_{k}$ is a 11 allergens by 6 time points matrix, for k=1,..,K, with K=176 subjects (See Section 3.2.1 for component number selection). For each component *r*, the allergen pattern ($a_{r}$), scaled subject-specific time profiles ($c_{k,r}{\left[b_{k}\right]}_{r}$), and the mean profile of scaled subject-specific time profiles are shown. In the last two columns, scaled subject-specific time profiles are colored according to the delivery/birth mode, i.e. natural birth (117 subjects), C-section (35 subjects) and vacuum extraction (24 subjects), and atopic disease diagnosis, i.e. ever diagnosed with asthma, atopic dermatitis (AD), and/or allergic rhinitis (AR) until 18 years old.

**Figure 6:**
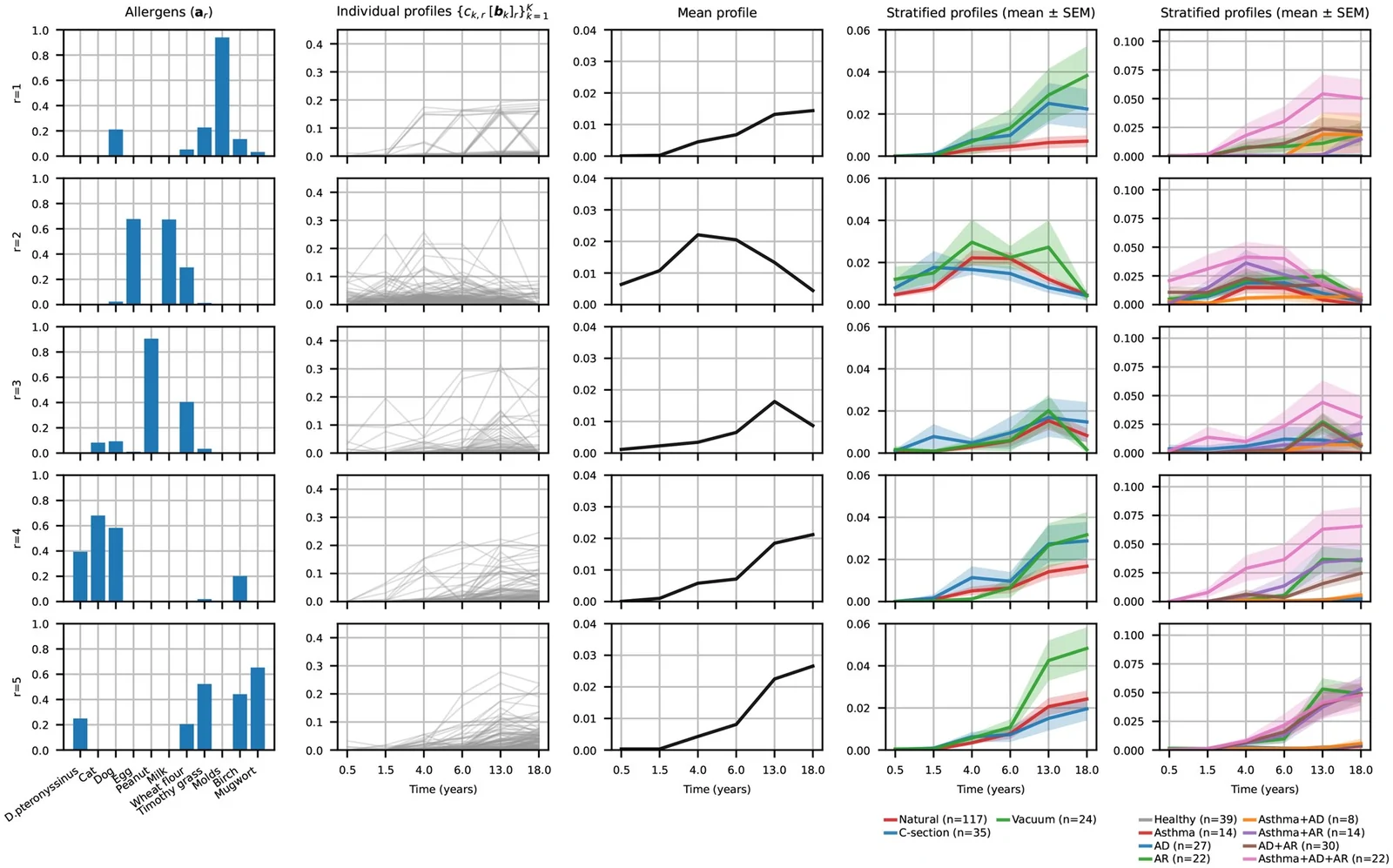
Sensitization. Components of a 5-component CMF model (with nonnegativity constraints in all modes) of the sensitization data. $a_{r}$ denotes the pattern in the allergens mode. Subject-specific time profiles scaled by the corresponding subject scores, i.e. $c_{k,r}{\left[b_{k}\right]}_{r}$, are shown in the second column. Mean of scaled subject-specific profiles are plotted in the third column. The rightmost columns show mean (and standard error of mean) of scaled subject-specific time profiles colored according to delivery/birth mode and disease groups.

Among the five components, two of them mainly model food allergens ($a_{2},a_{3}$), two of them model aeroallergens ($a_{1},a_{4}$), and one of them ($a_{5}$) contains allergens from both the groups. Component 2 models, in particular, the sensitization to egg, milk, and wheat flour. In the second column, we observe that individuals differ in terms of their sensitization to these allergens over time. In the third column, the average profile shows that, on average, the sensitization peaks around 4 years old and then decreases. In the fourth column, time profiles colored according to birth mode show different peaks at different ages for different groups—revealing group differences. Similarly, component 3 mainly models the sensitization to peanut (and also wheat flour), where in average sensitization peaks around 13 years old but also showing group differences in the fourth column. Component 1 primarily models mold (also dog, grass, and birch) with increasing sensitization over time in average. Component 4 models house dust mite, dog, cat, and, to some extent, birch sensitization also increasing over time. Finally, the last component models both mugworth, birch, grass, house dust mite, and also wheat flour showing increasing sensitization over time. In the last column, time profiles are colored according to whether subjects ever get diagnosed with asthma, AD, and/or AR or stay healthy—which shows that subject-specific sensitization profiles may also be studied to explore the link with diagnosis with one or more atopic diseases.

We compare the CMF model with a 5-component CP and 5-component PARAFAC2 model. All models reveal similar components in the allergens mode; however, they differ in terms of temporal trajectories. Figure 7 shows the component modeling the sensitization to egg, milk, and wheat flour extracted by each model (for all components, see Figs S.12 and S.13, available as supplementary data at *Bioinformatics* online). The CP model extracts the same temporal profile from each subject, only differing up to scaling. However, both CMF and PARAFAC2 can reveal shape differences in temporal profiles indicating subjects born by C-section showing earlier sensitization to these allergens than those born by natural birth. Similar differences emerge when stratifying by disease diagnosis (asthma, AD, and AR), e.g. in the peanut-dominated component, CMF, and PARAFAC2 uncover distinct trajectories across diagnosis groups that CP’s shared profile cannot capture.

**Figure 7:**
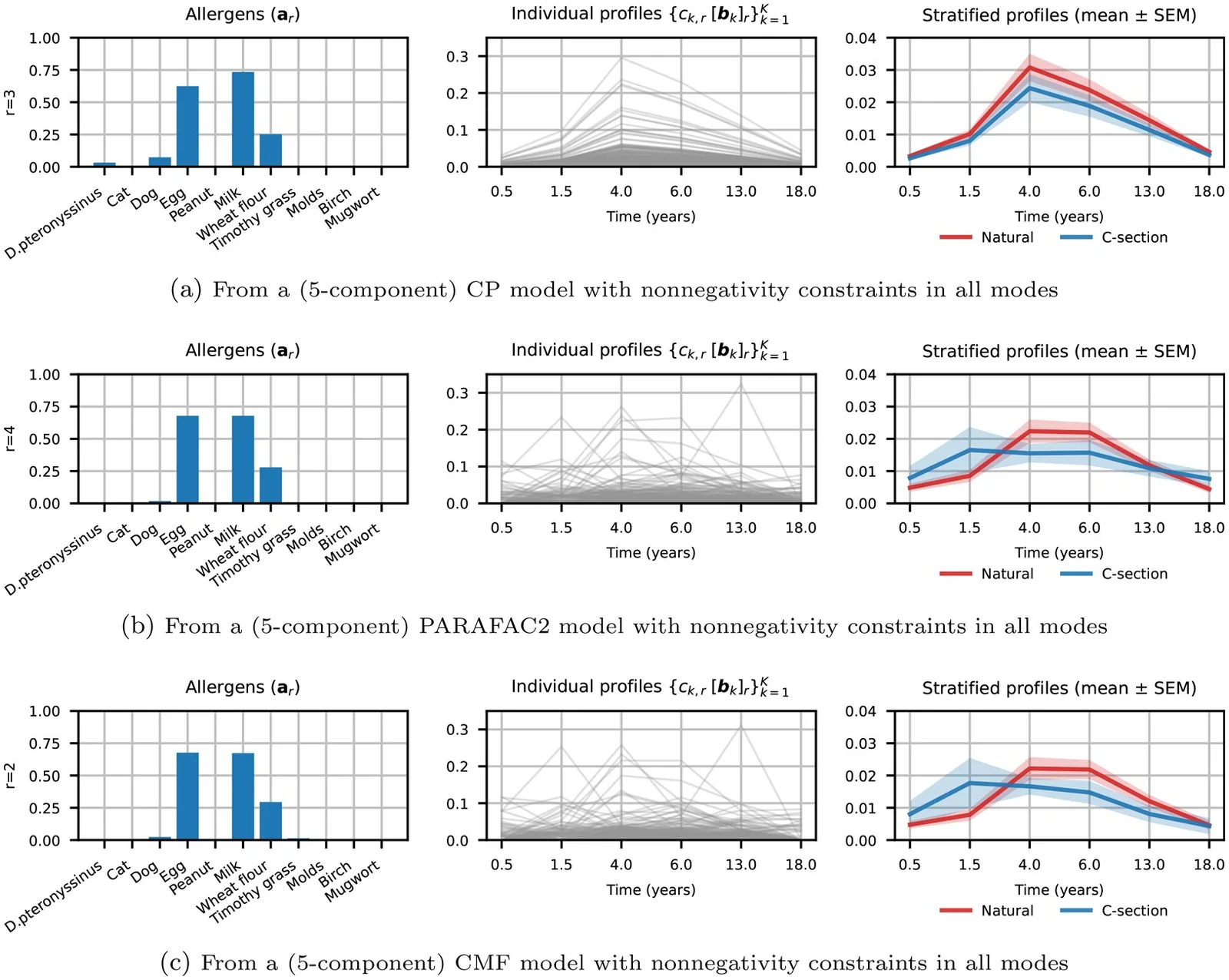
The food allergen (i.e. egg, milk, and wheat flour) dominated component extracted using different models. $a_{r}$ denotes the pattern in the allergens mode. Scaled subject-specific time profiles are shown in the middle column. The last column shows mean (and standard error of mean) patterns of scaled subject-specific time profiles colored according to delivery/birth mode. We only plot natural birth and C-section groups for clarity in the last column.

Compared to previous studies (Thorsen *et al.* 2025), CP reveals similar patterns such as components that model food allergens and their average time profiles, and components that model aeroallergens and their average time profile increasing over time. Due to sparsity constraints and the higher *R*, sparser components have previously been observed splitting some components that model aeroallergens (Thorsen *et al.* 2025). However, the overall interpretation remains the same, confirming previous findings. In addition, in this article, through the CMF and PARAFAC2 models, we reveal subject-specific sensitization trajectories showing that individuals have different temporal trajectories of sensitization and that the difference may be, for instance, related to past, e.g. the delivery mode, or may have potential links to future, e.g. disease diagnosis. Early-life risk factors for food allergies have previously been studied showing positive associations between cesarean delivery and food allergies, with an increased risk in the C-section group already when infants were less than 1 year old (Mitselou *et al.* 2018), and also in infants with allergic predisposition (Eggesbø *et al.* 2003). Our results are in line with these findings, and in addition, we provide more insights such as the allergen pattern with the specific food allergens as well as complete sensitization trajectories.

#### 3.2.1 Reproducibility and replicability

Figure 8a shows that we consistently obtain the same solution for all models for different *R*, except for PARAFAC2 when R=6 (in this case, no runs resulted in function values close to each other; therefore, 6-component PARAFAC2 was not included in the analysis). Figure 8b shows the replicability of patterns, using stratified subsets according to delivery mode. We observe that CMF and PARAFAC2 are more replicable than CP. We choose R=5 for CMF and PARAFAC2 based on both replicability and interpretation of the components. CMF is replicable at R=5 (for higher *R*, allergen components are splitted), and PARAFAC2 is replicable only up to R=5. For CP, with R=5, we get allergen components clustering allergens meaningfully as in other models; therefore, for comparisons we used the 5-component CP model but note that the model is not as replicable as other models.

**Figure 8:**
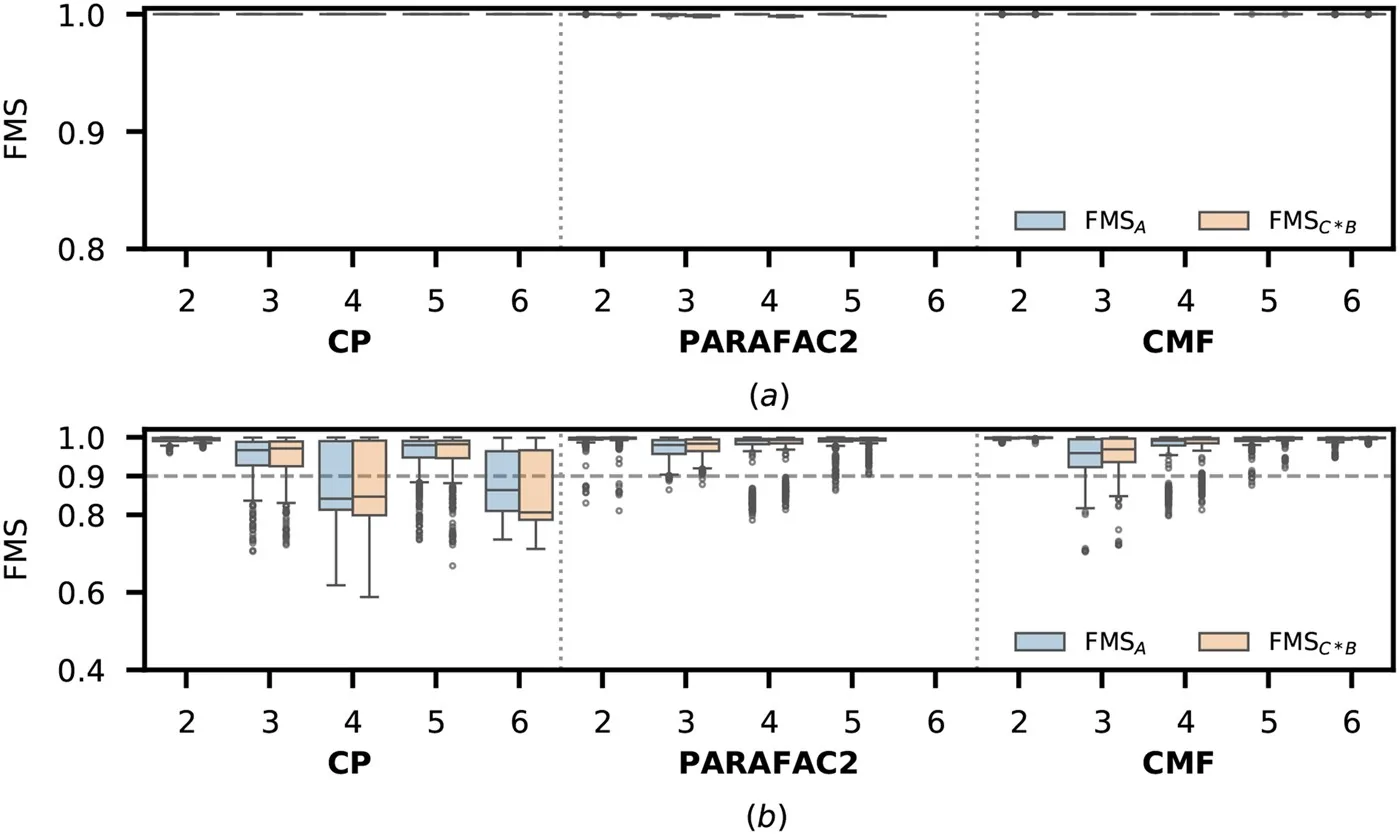
(a) Reproducibility and (b) replicability of different models of the sensitization data using different *R*.

## 4 Discussion

We observe that CMF and PARAFAC2 reveal similar components in terms of features (allergens or metabolites) and subject-specific time profiles in different applications. In the feature mode, these components are indeed quite similar as quantified in Table 1 (last row). However, in terms of subject-specific time profiles, while results look similar at the group level in Fig. 2 versus Fig. 4 for metabolomics data analysis, and in Fig. 7 for sensitization analysis, there are differences in scaled subject-specific time profiles. PARAFAC2 has an additional constraint, which comes with assumptions about orientations of components with respect to each other. When scaled subject-specific time profiles for each subject are compared, while the models (CMF versus PARAFAC2) agree well for the components we have highlighted, there are some components where the profiles for subjects do not match well (see Figs S.1 and S.14, available as supplementary data at *Bioinformatics* online). It has been previously demonstrated that if the PARAFAC2 constraint is significantly violated, then the model will fail to find the true patterns (Chatzis *et al.* 2023, 2025a). We have also explored this issue further by comparing CMF and PARAFAC2 on the task of uncovering patterns that do not conform to the constraint (Fig. S.16, available as supplementary data at *Bioinformatics* online) and demonstrate that CMF recovers the ground truth more accurately. This is consistent with the lower predictive performance of PARAFAC2-derived profiles relative to CMF when predicting BMI and HOMA-IR, suggesting that the cross-product constraint can distort subject-specific profiles in practice. Therefore, we consider CMF with nonnegativity constraints a better choice not to distort the time profiles with additional constraints. However, if the data is not nonnegative (due to centering or transformations (Erdos *et al.* 2026)), then one could still consider PARAFAC2.

**Table 1:** ${\text{FMS}}_{A}$ between CP, PARAFAC2, and CMF for both applications showing the similarity of components in the features mode.

	Metabolomics	Sensitization
CP versus CMF	0.95	0.99
CP versus PARAFAC2	0.98	0.99
CMF versus PARAFAC2	0.98	1.00

A common problem in longitudinal data analysis is missing samples. If subject *k* misses a sample at a time point, this corresponds to a missing column in $X_{k}$. In the metabolomics data, there are a few such subjects. In sensitization data, out of 411 subjects, there are 77 subjects missing one sample (i.e. one visit), 29, 28, 11, and four subjects missing two, three, four, and five samples, respectively. Missing columns are particularly challenging for CMF-based approaches including PARAFAC2 because such missing data results in non-unique models. In this study, we excluded anyone with missing samples for the patterns/findings not to be affected by missing data. We plan to address this issue via additional constraints such as temporal smoothness in future work.

In both applications, we observe that individual temporal profiles vary widely. To understand the main sources of variation among individual temporal profiles, we have looked into various groups of interest such as BMI/IR groups in metabolomics, and delivery mode and atopic disease diagnosis in sensitization data analysis. We have primarily relied on visualizations of mean and standard error of mean. However, there is high individual variation in subject-specific time profiles. Figs S.4 and S.15, available as supplementary data at *Bioinformatics* online, show how similar time profiles are among subjects for each method. Individual variation is relatively smaller in metabolomics data compared to sensitization data. While differences between stratified time profiles survive statistical tests for the metabolomics application, that is not the case for sensitization data analysis. Therefore, such relations with meta variables of interest should be validated on larger independent cohorts. Furthermore, a more systematic approach is needed to explore which meta variables should be of interest. One potential approach is to jointly analyse longitudinal data and other relevant data (e.g. variables of interest, other omics data) using coupled matrix and tensor factorizations (Schenker *et al.* 2025).

Subject-specific temporal profiles of the underlying patterns from longitudinal data are meaningful only when they can be extracted reliably. We have demonstrated both reproducibility and replicability of those patterns in our analysis. However, our replicability analysis has been limited since it relies on the replicability of patterns across subsets of the data. In other domains, it is possible to collect replicates from the same individuals, and assess replicability using those replicates such as in functional neuroimaging (Mørup *et al.* 2025). However, this approach remains a challenging issue in domains where measurements are expensive.

In conclusion, this article demonstrates that CMF-based methods are effective approaches to reliably capture subject-specific temporal patterns via two novel applications from metabolomics and sensitization data analysis. Our results show that those individual (shape) differences may reveal important insights, e.g. links with earlier exposures such as the delivery type or various phenotypes and diseases. We have assessed the reliability of the patterns considering both reproducibility and replicability. Validation of such insights using independent cohorts remains as future work.

## Supplementary Material

btag658_Supplementary_Data

## Data Availability

Clinical data are not publicly available due to privacy reasons. Data can be made available under a joint research collaboration by contacting COPSAC (administration@dbac.dk).

## References

[btag658-B1] Acar E , Yener B. Unsupervised multiway data analysis: a literature survey. IEEE Trans Knowl Data Eng 2009;21:6–20. 10.1109/TKDE.2008.112

[btag658-B2] Adali T , Kantar F, Akhonda MABS et al Reproducibility in matrix and tensor decompositions: focus on model match, interpretability, and uniqueness. IEEE Signal Process Mag 2022;39:8–24. 10.1109/msp.2022.3163870 PMC9635492 36337436

[btag658-B3] Amigo JM , Skov T, Bro R et al Solving GC–MS problems with PARAFAC2. Trends Anal Chem 2008;27:714–25. 10.1016/j.trac.2008.05.011

[btag658-B4] Badea L. Extracting gene expression profiles common to colon and pancreatic adenocarcinoma using simultaneous nonnegative matrix factorization. In: *Pacific Symp. on Biocomputing,* Kohala Coast, Hawaii, USA: World Scientific, 2008, 279–90. 18229692

[btag658-B5] Ballard G , Kolda TG. Tensor Decompositions for Data Science. Cambridge University Press, 2025.

[btag658-B6] Berry SE , Valdes AM, Drew DA et al Human postprandial responses to food and potential for precision nutrition. Nat Med 2020;26:964–73. 10.1038/s41591-020-0934-0 PMC8265154 32528151

[btag658-B7] Bisgaard H. The Copenhagen Prospective Study on Asthma in Childhood (COPSAC): design, rationale, and baseline data from a longitudinal birth cohort study. Ann Allergy Asthma Immunol 2004;93:381–9. 10.1016/S1081-1206(10)61398-1 15521375

[btag658-B8] Carroll JD , Chang J-J. Analysis of individual differences in multidimensional scaling via an *n*-way generalization of “Eckart–Young” decomposition. Psychometrika 1970;35:283–319. 10.1007/BF02310791

[btag658-B9] Chatzis C , Pfeffer M, Lind P et al A time-aware tensor decomposition for tracking evolving patterns. In: *IEEE 33rd International Workshop on Machine Learning for Signal Processing (MLSP)*. Rome, Italy: IEEE, 2023, 1–6.

[btag658-B10] Chatzis C , Schenker C, Cohen JE et al dCMF: learning interpretable evolving patterns from temporal multiway data. In: 33rd European Signal Processing Conference (EUSIPCO). Palermo, Italy: IEEE, 2025a, 2122–6.

[btag658-B11] Chatzis C , Schenker C, Pfeffer M et al tPARAFAC2: tracking evolving patterns in (incomplete) temporal data. Data Min Knowl Disc 2025b;39:83. 10.1007/s10618-025-01122-6

[btag658-B12] Eggesbø M , Botten G, Stigum H et al Is delivery by cesarean section a risk factor for food allergy? J Allergy Clin Immunol 2003;112:420–6. 10.1067/mai.2003.1610 12897751

[btag658-B13] Erdos B et al Extracting host-specific developmental signatures from longitudinal microbiome data. PLoS Comput Biol 2026;22:1–21. 10.1371/journal.pcbi.1014486 PMC13362426 42406847

[btag658-B14] Harshman RA. Foundations of the PARAFAC procedure: models and conditions for an “explanatory” multi-modal factor analysis. UCLA Working Papers in Phonetics 1970;16:1–84.

[btag658-B15] Harshman RA. PARAFAC2: mathematical and technical notes. UCLA Working Papers in Phonetics 1972;22:30–47.

[btag658-B16] Jansen JJ , Szymańska E, Hoefsloot HCJ et al Individual differences in metabolomics: individualised responses and between-metabolite relationships. Metabolomics 2012;8:94–104. 10.1007/s11306-012-0414-8 PMC3337417 22593723

[btag658-B17] Kiers H , Ten Berge J, Bro R. PARAFAC2—part I. A direct fitting algorithm for the PARAFAC2 model. J Chemometrics 1999;13:275–94. 10.1002/(SICI)1099-128X(199905/08)13:3/4<275::AID-CEM543>3.0.CO; 2-B

[btag658-B18] Kim JY , Michaliszyn SF, Nasr A et al The shape of the glucose response curve during an oral glucose tolerance test heralds biomarkers of type 2 diabetes risk in obese youth. Diabetes Care 2016;39:1431–9. 10.2337/dc16-0352 PMC4955931 27293201

[btag658-B19] Kossaifi J et al TensorLy: tensor learning in python. J Mach Learn Res 2019;20:1–6.

[btag658-B20] Kruskal JB. Three-way arrays: rank and uniqueness of trilinear decompositions, with application to arithmetic complexity and statistics. Linear Algebra Appl 1977;18:95–138. 10.1016/0024-3795(77)90069-6

[btag658-B21] Li L , Yan S, Horner D et al Revealing static and dynamic biomarkers from postprandial metabolomics data through coupled matrix and tensor factorizations. Metabolomics 2024;20:86. 10.1007/s11306-024-02128-9 39066850

[btag658-B22] Ma S , Li H. A tensor decomposition model for longitudinal microbiome studies. Ann Appl Stat 2023;17:1105–26. 10.1214/22-AOAS1661

[btag658-B23] Madsen KH , Churchill NW, Mørup M. Quantifying functional connectivity in multi-subject fMRI data using component models: quantifying functional connectivity. Hum Brain Mapp 2017;38:882–99. 10.1002/hbm.23425 PMC6866892 27739635

[btag658-B24] Martino C , Shenhav L, Marotz CA et al Context-aware dimensionality reduction deconvolutes gut microbial community dynamics. Nat Biotechnol 2021;39:165–8. 10.1038/s41587-020-0660-7 PMC7878194 32868914

[btag658-B25] Metwally AA , Zhang T, Wu S et al Robust identification of temporal biomarkers in longitudinal omics studies. Bioinformatics 2022;38:3802–11. 10.1093/bioinformatics/btac403 PMC9344853 35762936

[btag658-B26] Mitselou N , Hallberg J, Stephansson O et al Cesarean delivery, preterm birth, and risk of food allergy: nationwide Swedish cohort study of more than 1 million children. J Allergy Clin Immunol 2018;142:1510–4.e2. 10.1016/j.jaci.2018.06.044 30213656

[btag658-B27] Mørup M , Acar E, Adali T. Tensor and coupled decompositions for interpretable pattern discovery in multimodal functional neuroimaging data. IEEE Signal Process Mag 2025;42:41–57. 10.1109/MSP.2025.3605746

[btag658-B28] Perros I , Papalexakis EE, Vuduc R et al Temporal phenotyping of medically complex children via PARAFAC2 tensor factorization. J Biomed Inform 2019;93:103125. 10.1016/j.jbi.2019.103125 30743070

[btag658-B29] Roald M. MatCoupLy: learning coupled matrix factorizations with python. SoftwareX 2023;21:101292. 10.1016/j.softx.2022.101292

[btag658-B30] Roald M , Schenker C, Calhoun VD et al An AO-ADMM approac to constraining PARAFAC2 on all modes. SIAM J Math Data Sci 2022;4:1191–222. 10.1137/21M1450033

[btag658-B31] Schenker C , Wang X, Horner D et al PARAFAC2-based coupled matrix and tensor factorizations with constraints. IEEE J Sel Top Signal Process 2025;19:1461–76. 10.1109/JSTSP.2025.3579651

[btag658-B32] Schoos A-MM , Chawes BL, Melén E et al Sensitization trajectories in childhood revealed by using a cluster analysis. J Allergy Clin Immunol 2017;140:1693–9. 10.1016/j.jaci.2017.01.041 28347735

[btag658-B33] Schoos A-MM , Hansen SM, Skov FR et al Allergen specificity in specific IgE cutoff. JAMA Pediatr 2020;174:993–5. 10.1001/jamapediatrics.2020.0944 PMC7235915 32421160

[btag658-B34] Shi P , Martino C, Han R et al TEMPTED: time-informed dimensionality reduction for longitudinal microbiome studies. Genome Biol 2024;25:317. 10.1186/s13059-024-03453-x PMC11657513 39696594

[btag658-B35] Silva CDCD , Zambon MP, Vasques ACJ et al The threshold value for identifying insulin resistance (HOMA-IR) in an admixed adolescent population: a hyperglycemic clamp validated study. Arch Endocrinol Metab 2022. 10.20945/2359-3997000000533 PMC9983787 36468919

[btag658-B36] Singh AP , Gordon GJ. Relational learning via collective matrix factorization. In: *Proceedings of the 14th ACM SIGKDD International Conference Knowledge Discovery and Data Mining*. Las Vegas, NV, USA: ACM; 2008, 650–8.

[btag658-B37] Siroux V , Boudier A, Bousquet J et al Trajectories of IgE sensitization to allergen molecules from childhood to adulthood and respiratory health in the EGEA cohort. Allergy 2022;77:609–18. 10.1111/all.14987 34169532

[btag658-B38] Smilde A , Bro R, Geladi P. Multi-Way Analysis: Applications in the Chemical Sciences. West Sussex, England: Wiley, 2004.

[btag658-B39] Thorsen J , Rasmussen MA, Chawes B et al Longitudinal sensitization patterns in childhood and adolescence. Allergy 2025;80:893–5. 10.1111/all.16408 39601622

[btag658-B40] van der Ploeg GR , Westerhuis JA, Heintz-Buschart A et al parafac4microbiome: exploratory analysis of longitudinal microbiome data using parallel factor analysis. mSystems 2025;10:e00472–25. 10.1128/msystems.00472-25 PMC12172464 40396737

[btag658-B41] Wopereis S. Phenotypic flexibility in nutrition research to quantify human variability: building the bridge to personalised nutrition. Proc Nutr Soc 2023;82:346–58. 10.1017/S0029665122002853 36503652

[btag658-B42] Yan S , Li L, Horner D et al Characterizing human postprandial metabolic response using multiway data analysis. Metabolomics 2024;20:50. 10.1007/s11306-024-02109-y PMC11082008 38722393

[btag658-B43] Zhou J et al From micro to macro: data driven phenotyping by densification of longitudinal electronic medical records. In: *Proceedings of the 20th ACM SIGKDD International Conference Knowledge Discovery and Data Mining*. New York, NY, USA: ACM; 2014, 135–44.

