# Supplementary material for: Revealing subject-specific temporal patterns from longitudinal data
Source: Bioinformatics. 2026 Sep 4;42(9):btag658. doi: 10.1093/bioinformatics/btag658 (PMC13623513; doi:10.1093/bioinformatics/btag658)
Supplement: Supplementary file 1 [file btag658_supplementary_data.pdf]

Supplementary Material for "Revealing Subject-Specific Temporal Patterns from Longitudinal Data"

Christos Chatzis[1,2*], David Horner[3], Rasmus Bro[4], Ann-Marie Malby Schoos[3,5,6,7], Morten A. Rasmussen[3,4], and Evrim Acar[1*]

[1]Department of Data Science and Knowledge Discovery, Simula Metropolitan Center for Digital Engineering, Oslo, Norway

[2]Faculty of Technology, Art and Design, Oslo Metropolitan University, Oslo, Norway

[3]COPSAC, Copenhagen Prospective Studies on Asthma in Childhood, Herlev and Gentofte Hospital, University of Copenhagen, Copenhagen, Denmark

[4]Department of Food Science, University of Copenhagen, Copenhagen, Denmark

[5]Department of Pediatrics, Copenhagen University Hospital - Næstved, Slagelse and Ringsted, Slagelse, Denmark

[6]Department of Pediatrics, Copenhagen University Hospital - Amager and Hvidovre, Hvidovre, Denmark

[7]Department of Clinical Medicine, University of Copenhagen, Copenhagen, Denmark

[*]Corresponding authors: christos@simula.no (C.C.) and evrim@simula.no (E.A.).

1 Metabolomics Data Analysis

1.1 Males

Table 1: Adjusted R^2 per component and method for predicting `BMI`. Best values of each method are in bold, which all correspond to the insulin/c-peptide-dominated component.

	**CP**	**CMF**	**PARAFAC2**
Component 1	0.06	0.25	0.22
Component 2	0.08	0.19	0.15
Component 3	0.15	0.21	**0.39**
Component 4	-0.06	0.32	0.21
Component 5	-0.05	0.04	0.05
Component 6	**0.23**	**0.41**	0.009

Table 2: Adjusted R^2 per component and method for predicting `HOMAIR`. Best values of each method are in bold, which all correspond to the insulin/c-peptide-dominated component.

	**CP**	**CMF**	**PARAFAC2**
Component 1	0.03	0.22	0.23
Component 2	0.17	0.25	0.25
Component 3	0.11	0.28	**0.28**
Component 4	-0.02	0.23	0.23
Component 5	-0.06	0.07	0.08
Component 6	**0.21**	**0.32**	0.06

Table 3: CMF/Males only. Top five metabolites per component with p-values showing the association between subject scores and lower/higher BMI groups.

**Component**	**Top 5 Metabolites**	**p-value**	**Sig.**
1	M-VLDL-CE, M-VLDL-C, M-VLDL-FC, S-VLDL-FC, S-VLDL-C	0.0183	*
2	L-HDL-FC, XL-HDL-PL, L-HDL-P, L-HDL-C, L-HDL-CE	0.0001	***
3	XXL-VLDL-PL, XXL-VLDL-P, XXL-VLDL-TG, XXL-VLDL-L, XXL-VLDL-FC	0.0009	***
4	Gly, Acetate, Leu, Ile, Total BCAA	0.0502	ns
5	bOHbutyrate, Acetoacetate, Acetone, Acetate, Leu	0.7699	ns
6	Insulin, Cpeptid, Glucose, Ile, Leu	0.0000	***

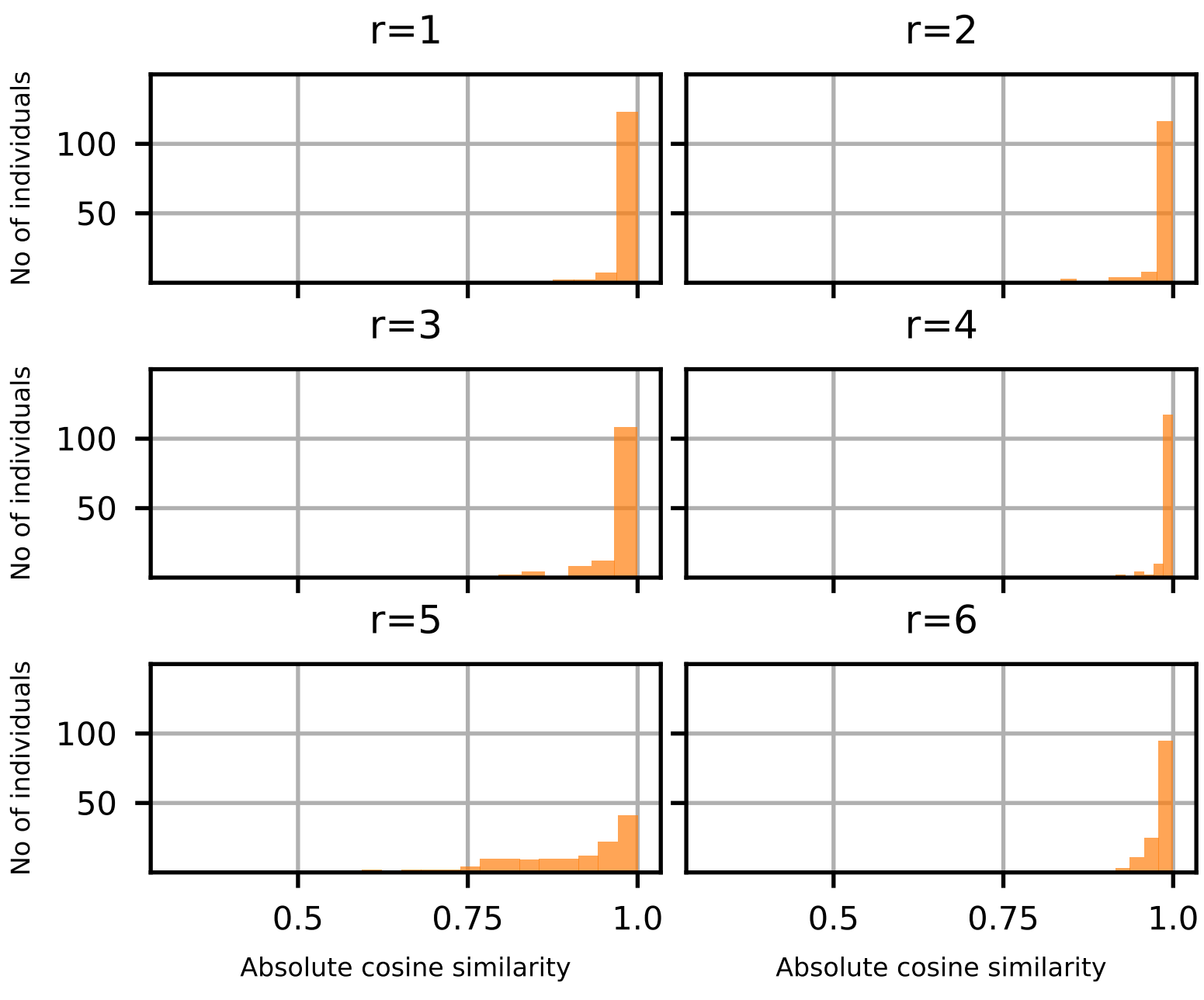

Figure S.1: Males. Comparison of subject-specific time profiles ($c_{k,r}[\mathbf{b}_k]_r$) captured by CMF and PARAFAC2 for each component for the metabolomics data. Each histogram contains 140 data points, one for each subject. The order is adjusted (following the order in the CMF model) so that matching components from different models are compared.

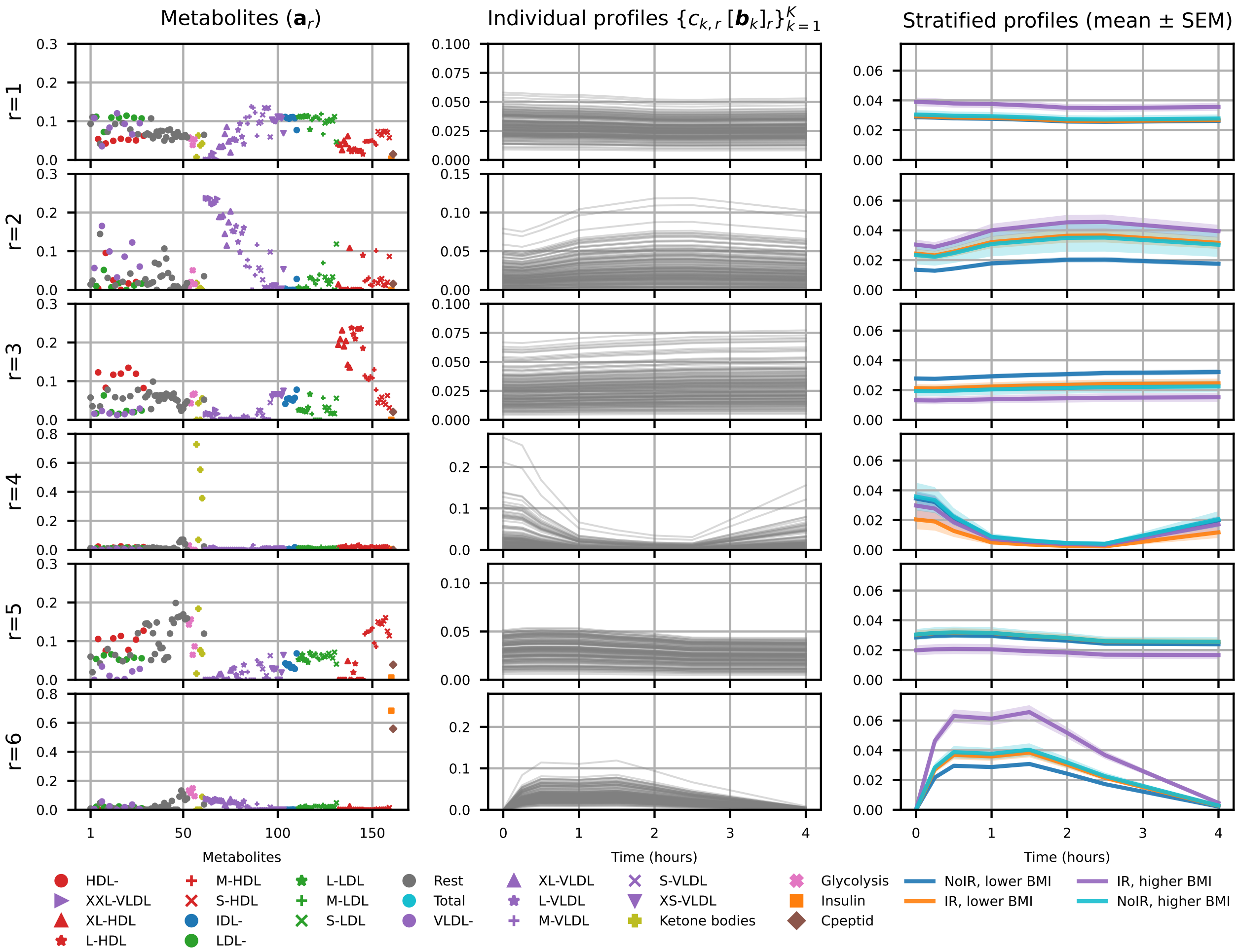

Figure S.2: Males. Components of a 6-component CP model (with nonnegativity constraints in all modes). $\mathbf{a}_r$ denotes the pattern in the metabolites mode, where metabolites are colored by lipoprotein classes. Different shapes are used for lipoprotein subclasses. Subject-specific time profiles scaled by the corresponding subject scores, i.e., $c_{k,r}[\mathbf{b}_k]_r$ for each component are shown in the middle column. The last column shows scaled subject-specific time profiles colored according to four BMI/IR groups.

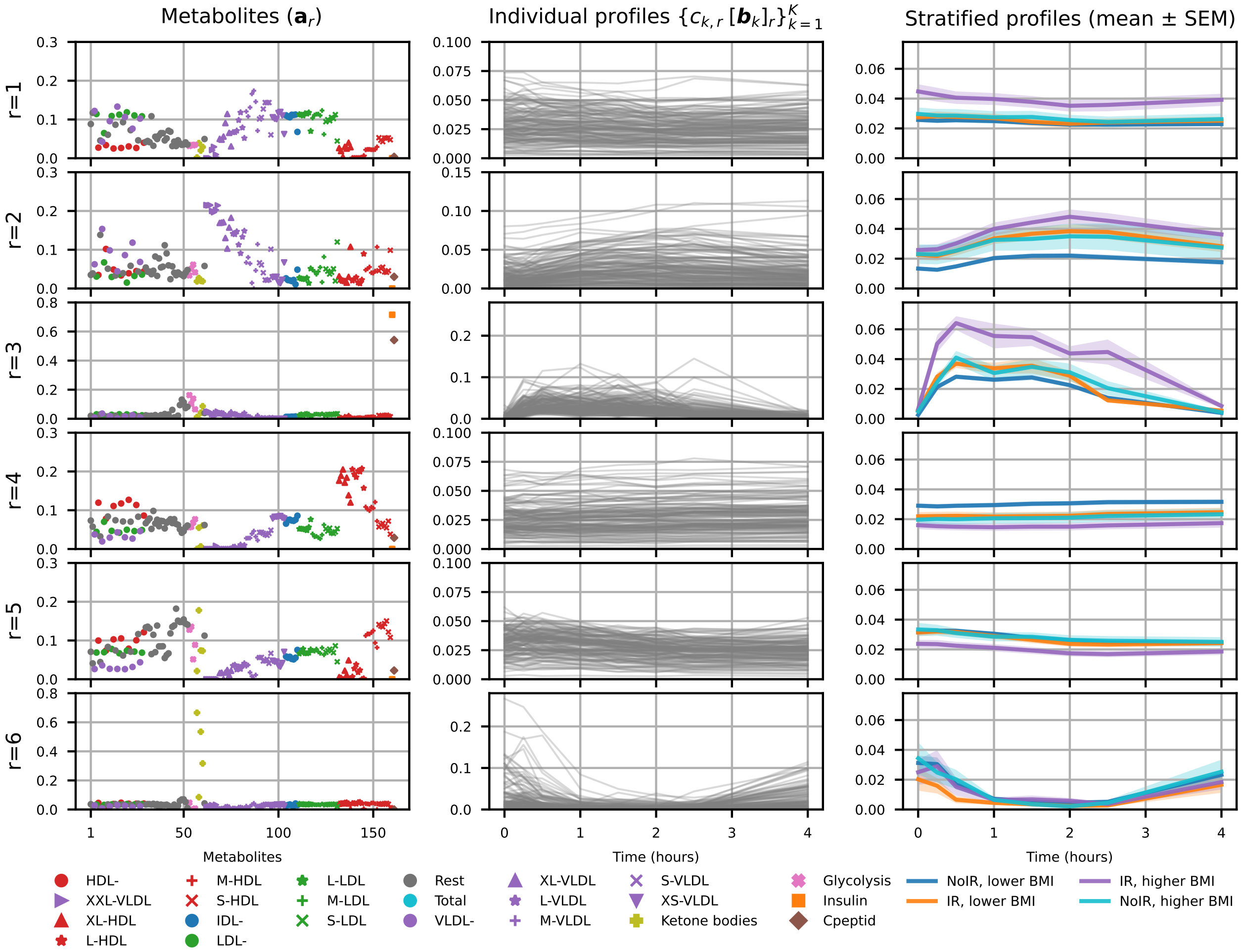

Figure S.3: Males. Components of a 6-component PARAFAC2 model (with nonnegativity constraints in all modes). $\mathbf{a}_r$ denotes the pattern in the metabolites mode, where metabolites are colored by lipoprotein classes. Different shapes are used for lipoprotein subclasses. Subject-specific time profiles scaled by the corresponding subject scores, i.e., $c_{k,r}[\mathbf{b}_k]_r$ for each component are shown in the middle column. The last column shows scaled subject-specific time profiles colored according to four BMI/IR groups.

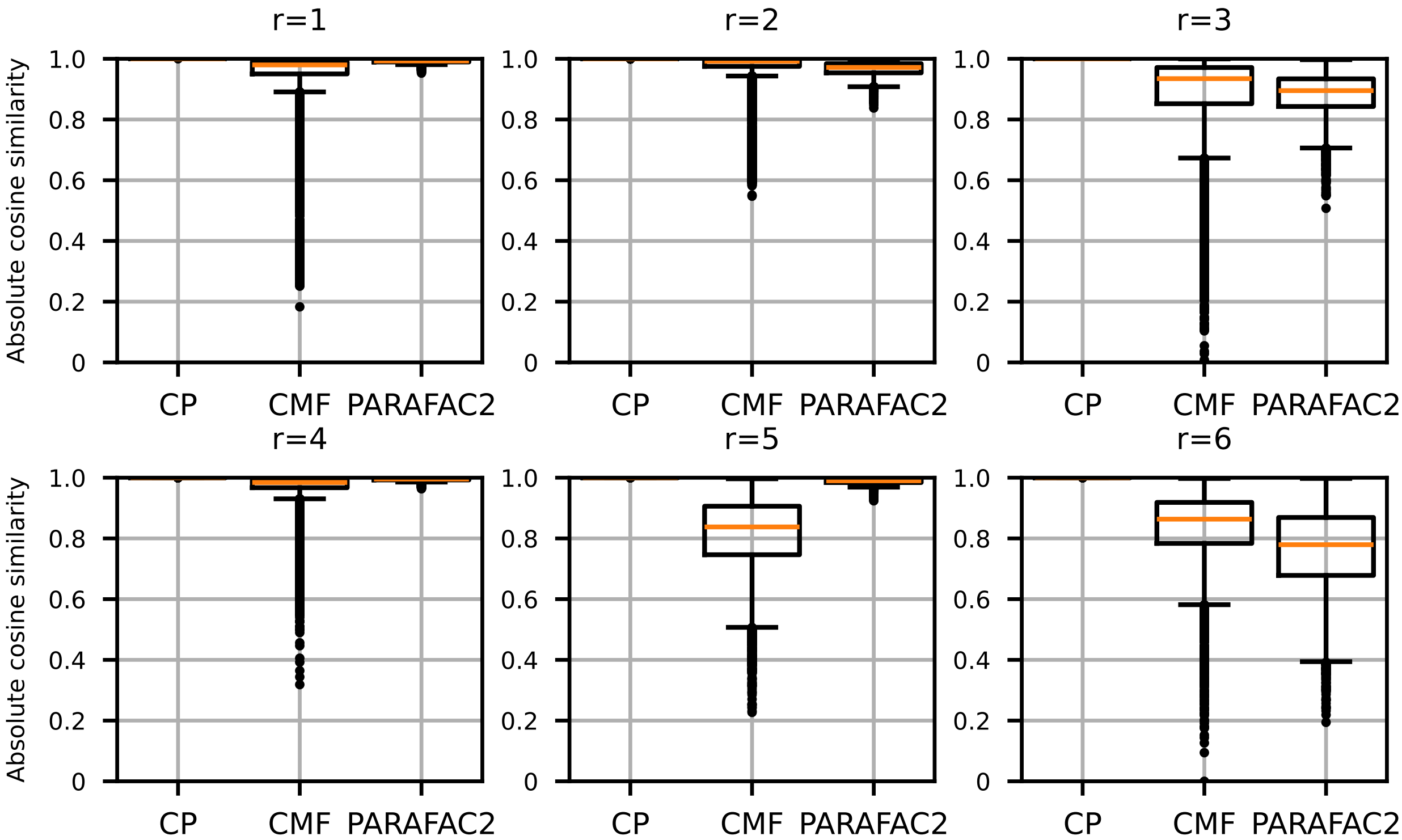

Figure S.4: Comparison of subject-specific time profiles in the metabolomics application (males only) within each component for each method in terms of absolute cosine similarity. The ordering of the components is determined by CMF, i.e. Fig. 2, and CP and PARAFAC2 are permuted so they match this exact order. CP does not allow structural variability, therefore the similarity between profiles is always one.

1.2 Females

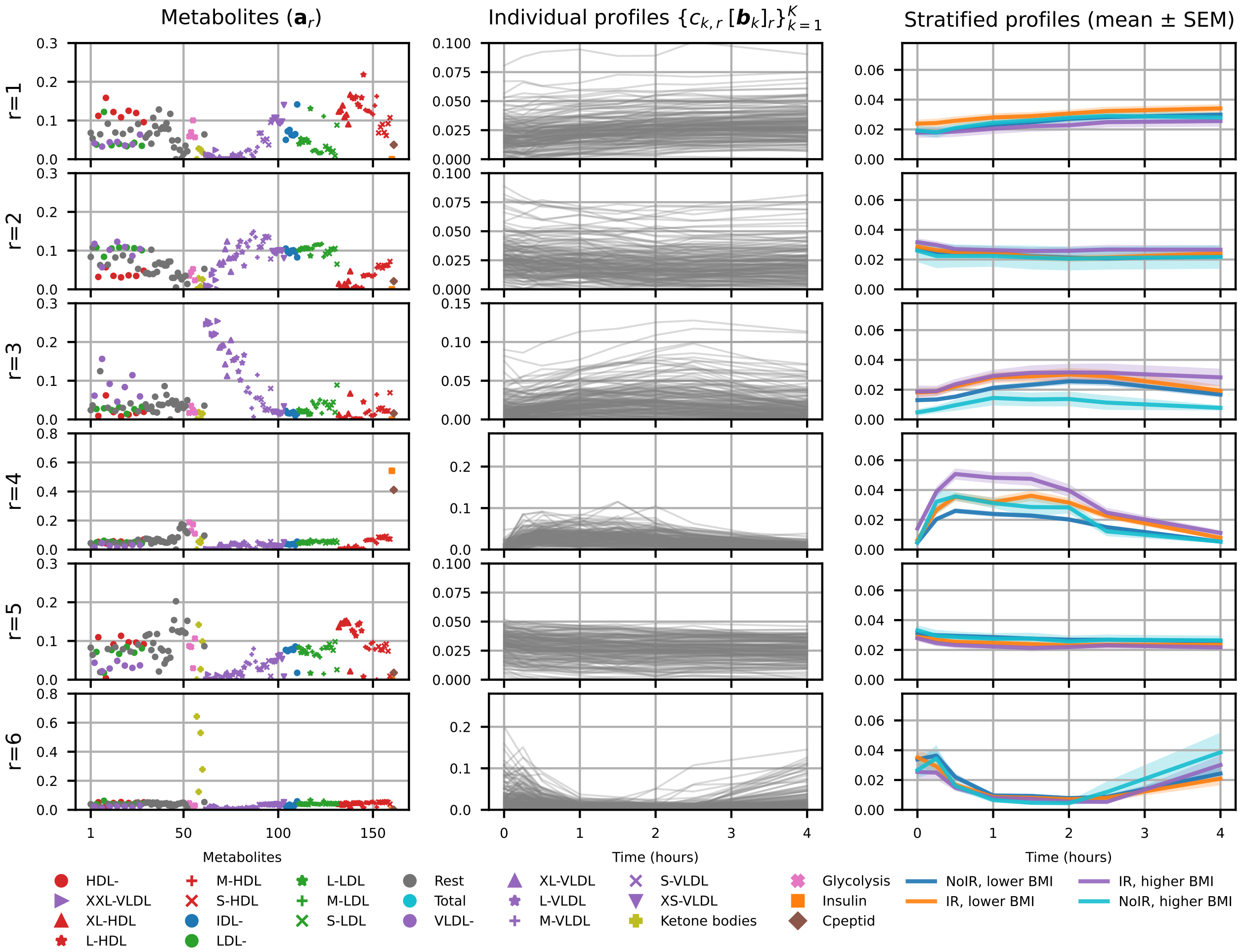

Figure S.5: Females. Components of a 6-component CMF model (with nonnegativity constraints in all modes). $\mathbf{a}_r$ denotes the pattern in the metabolites mode, where metabolites are colored by lipoprotein classes. Different shapes are used for lipoprotein subclasses. Subject-specific time profiles scaled by the corresponding subject scores, i.e., $c_{k,r}[\mathbf{b}_k]_r$ for each component are shown in the middle column. The last column shows scaled subject-specific time profiles colored according to four BMI/IR groups.

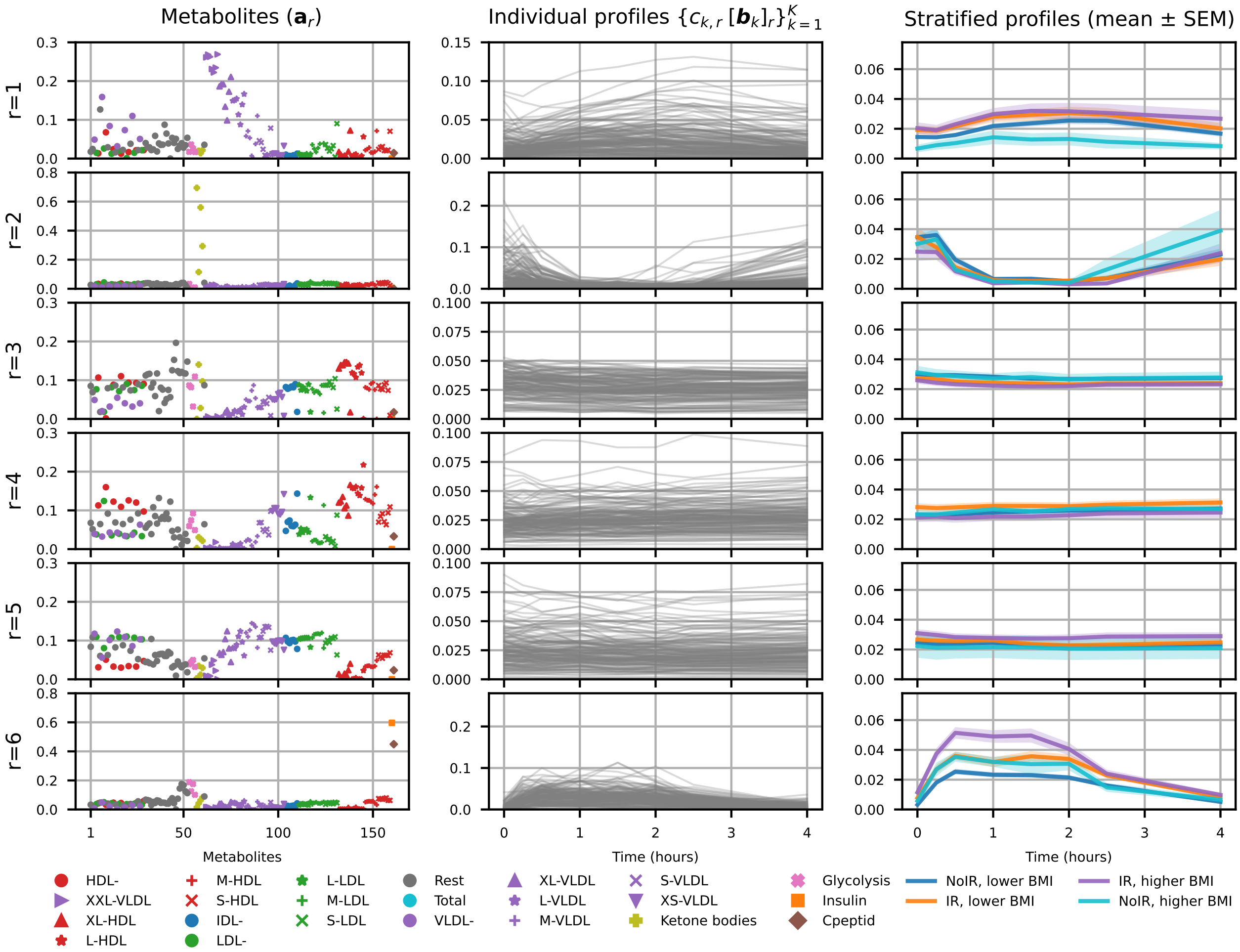

Figure S.6: Females. Components of a 6-component PARAFAC2 model (with nonnegativity constraints in all modes). $\mathbf{a}_r$ denotes the pattern in the metabolites mode, where metabolites are colored by lipoprotein classes. Different shapes are used for lipoprotein subclasses. Subject-specific time profiles scaled by the corresponding subject scores, i.e., $c_{k,r}[\mathbf{b}_k]_r$ for each component are shown in the middle column. The last column shows scaled subject-specific time profiles colored according to four BMI/IR groups.

Table 4: CMF/Females only. Top five metabolites per component with p-values showing the association between subject scores and lower/higher BMI groups.

Component	Top 5 Metabolites	p-value	Sig.
1	L-HDL-TG, XL-HDL-TG, M-HDL-TG, L-HDL-PL, L-HDL-P	0.4368	ns
2	M-VLDL-CE, M-VLDL-C, S-VLDL-CE, S-VLDL-C, M-VLDL-FC	0.3400	ns
3	XXL-VLDL-TG, XXL-VLDL-L, XXL-VLDL-PL, XXL-VLDL-P, XXL-VLDL-FC	0.7040	ns
4	Insulin, Cpeptid, Glucose, Ile, Pyruvate	0.0000	***
5	Gly, Gln, XL-HDL-CE, Tyr, XL-HDL-C	0.0695	ns
6	bOHbutyrate, Acetoacetate, Acetone, Acetate, L-LDL-TG	0.3747	ns

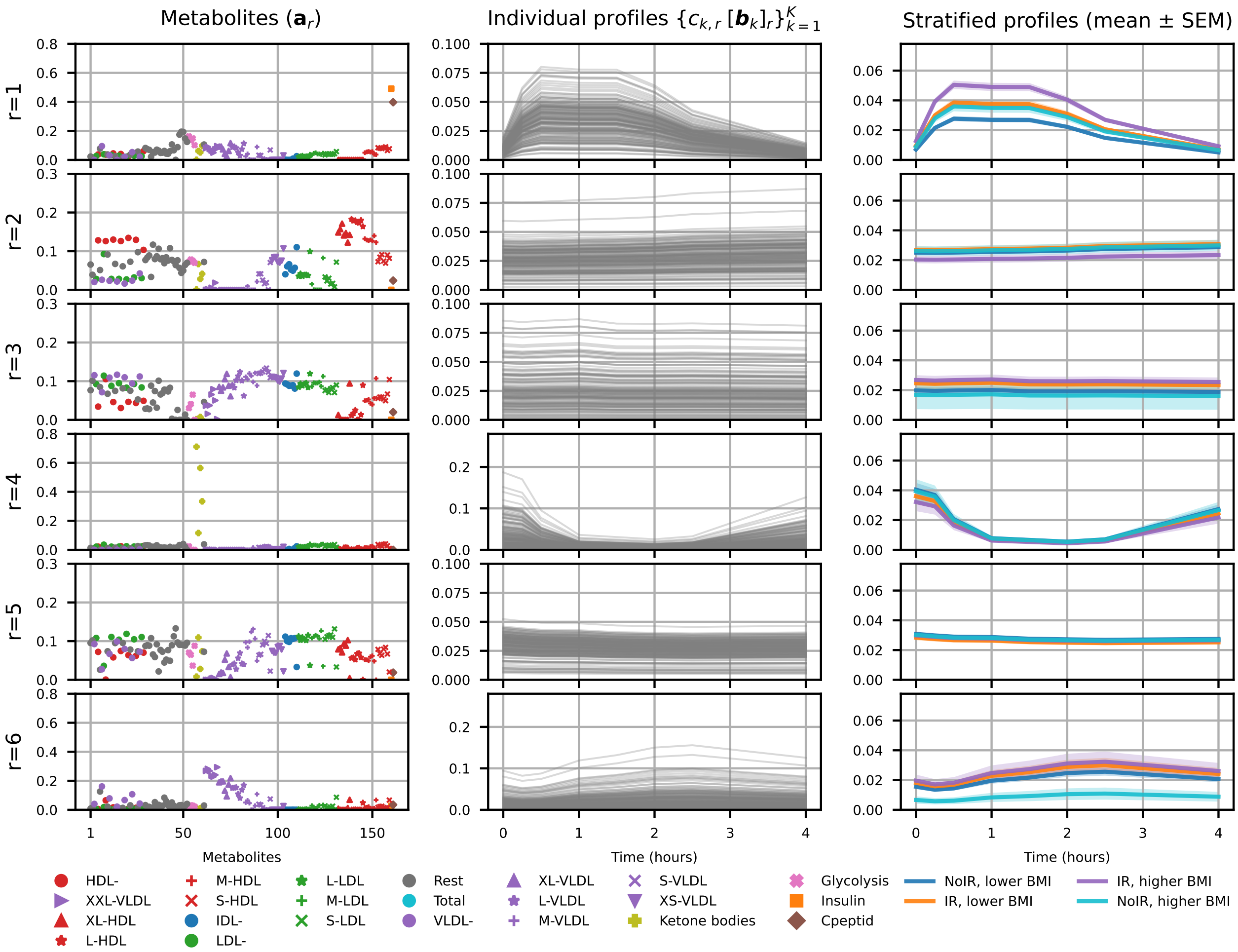

Figure S.7: Females. Components of a 6-component CP model (with nonnegativity constraints in all modes). $\mathbf{a}_r$ denotes the pattern in the metabolites mode, where metabolites are colored by lipoprotein classes. Different shapes are used for lipoprotein subclasses. Subject-specific time profiles scaled by the corresponding subject scores, i.e., $c_{k,r}[\mathbf{b}_k]_r$ for each component are shown in the middle column. The last column shows scaled subject-specific time profiles colored according to four BMI/IR groups.

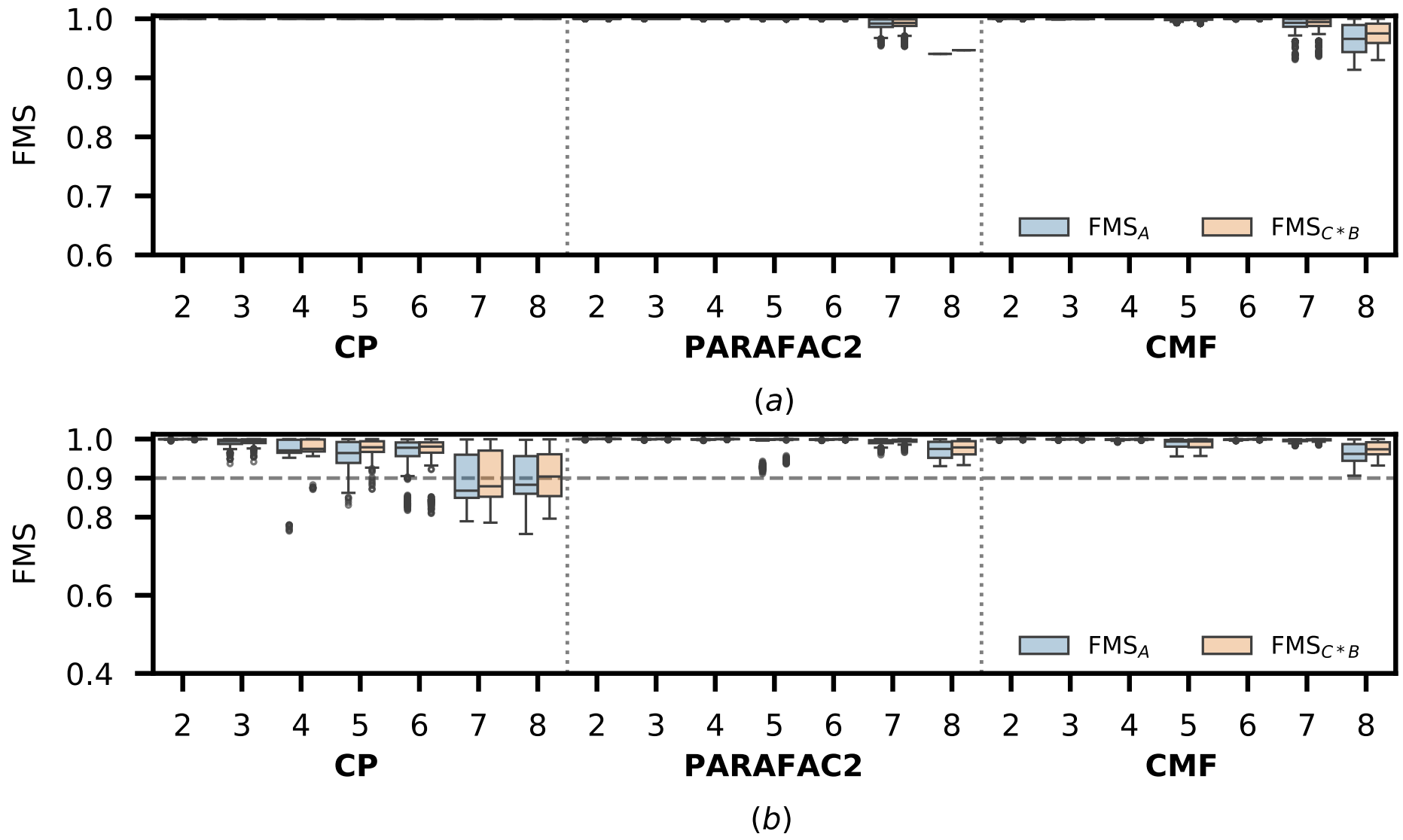

Figure S.8: Females. (a) Reproducibility, and (b) Replicability of different models of the metabolomics data using different number of components (R).

1.3 Males and Females

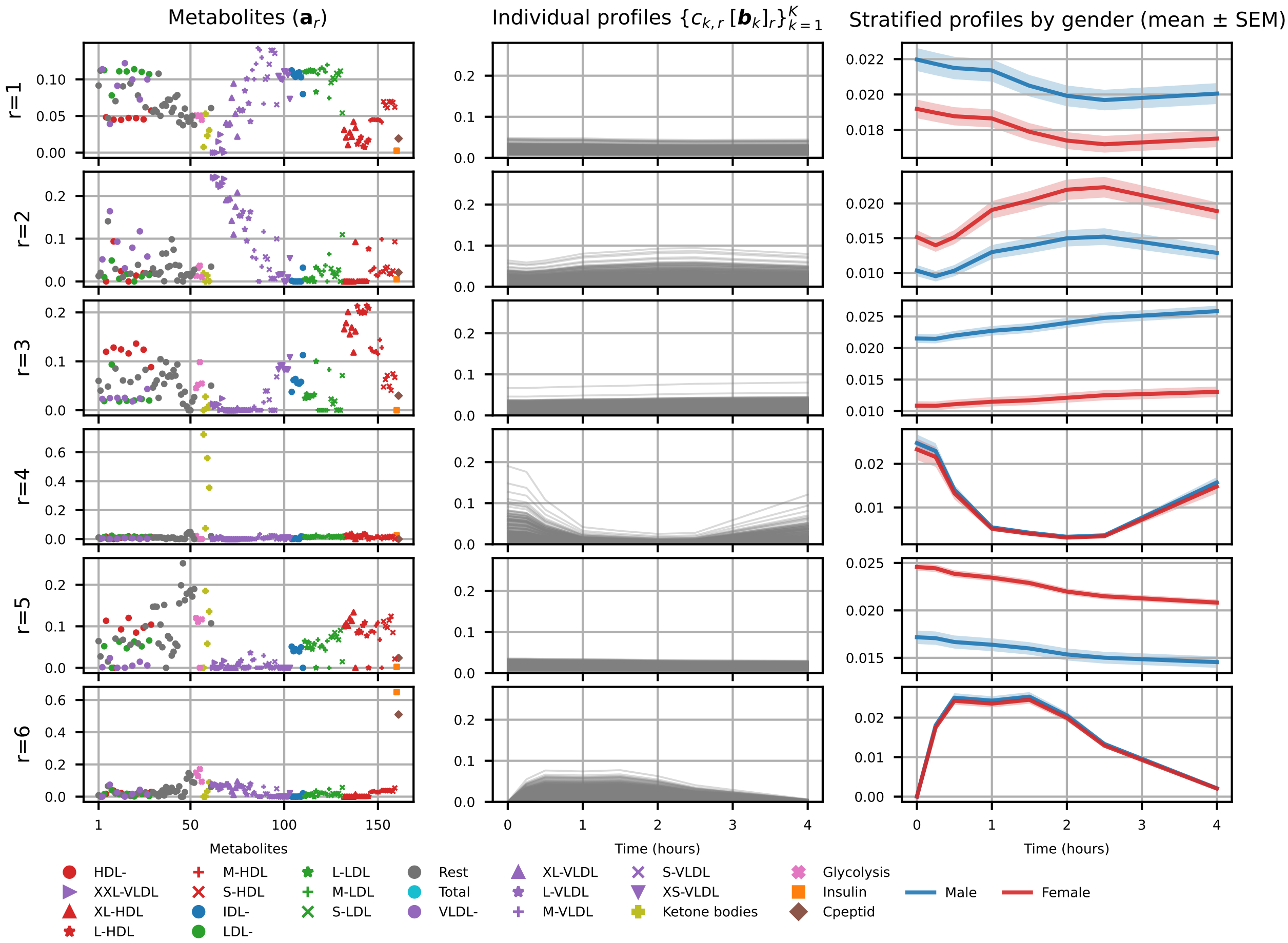

Figure S.9: Males and females. Components of a 6-component CP model (with nonnegativity constraints in all modes). $\mathbf{a}_r$ denotes the pattern in the metabolites mode, where metabolites are colored by lipoprotein classes. Different shapes are used for lipoprotein subclasses. Subject-specific time profiles scaled by the corresponding subject scores, i.e., $c_{k,r}[\mathbf{b}_k]_r$ for each component are shown in the middle column. The last column shows scaled subject-specific time profiles colored according to gender.

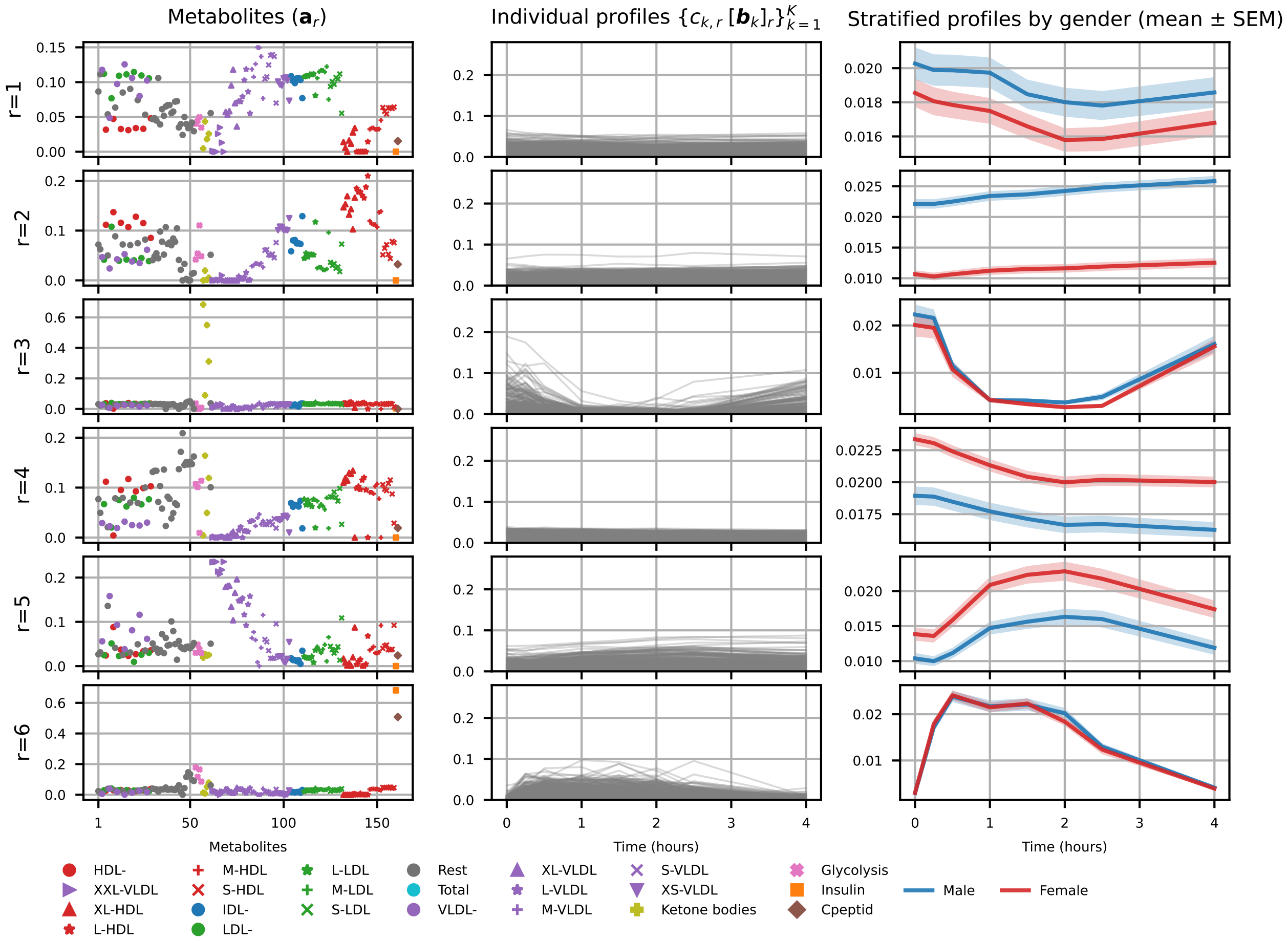

Figure S.10: Males and females. Components of a 6-component PARAFAC2 model (with nonnegativity constraints in all modes). $\mathbf{a}_r$ denotes the pattern in the metabolites mode, where metabolites are colored by lipoprotein classes. Different shapes are used for lipoprotein subclasses. Subject-specific time profiles scaled by the corresponding subject scores, i.e., $c_{k,r}[\mathbf{b}_k]_r$ for each component are shown in the middle column. The last column shows scaled subject-specific time profiles colored according to gender.

Table 5: CMF/Males and Females. Top five metabolites per component with p-values showing the association between subject scores and lower/higher BMI groups.

Component	Top 5 Metabolites	p-value	Sig.
1	L-HDL-TG, L-HDL-FC, L-HDL-P, L-HDL-L, L-HDL-PL	0.0298	*
2	bOHbutyrate, Acetoacetate, Acetone, Acetate, Leu	0.6397	ns
3	Insulin, Cpeptid, Glucose, Pyruvate, Ile	0.0000	***
4	XXL-VLDL-TG, XXL-VLDL-L, XXL-VLDL-PL, XXL-VLDL-P, XXL-VLDL-FC	0.0068	**
5	Gly, Gln, Tyr, Acetate, Leu	0.0002	***
6	M-VLDL-CE, M-VLDL-C, S-VLDL-CE, S-VLDL-C, M-VLDL-FC	0.0224	*

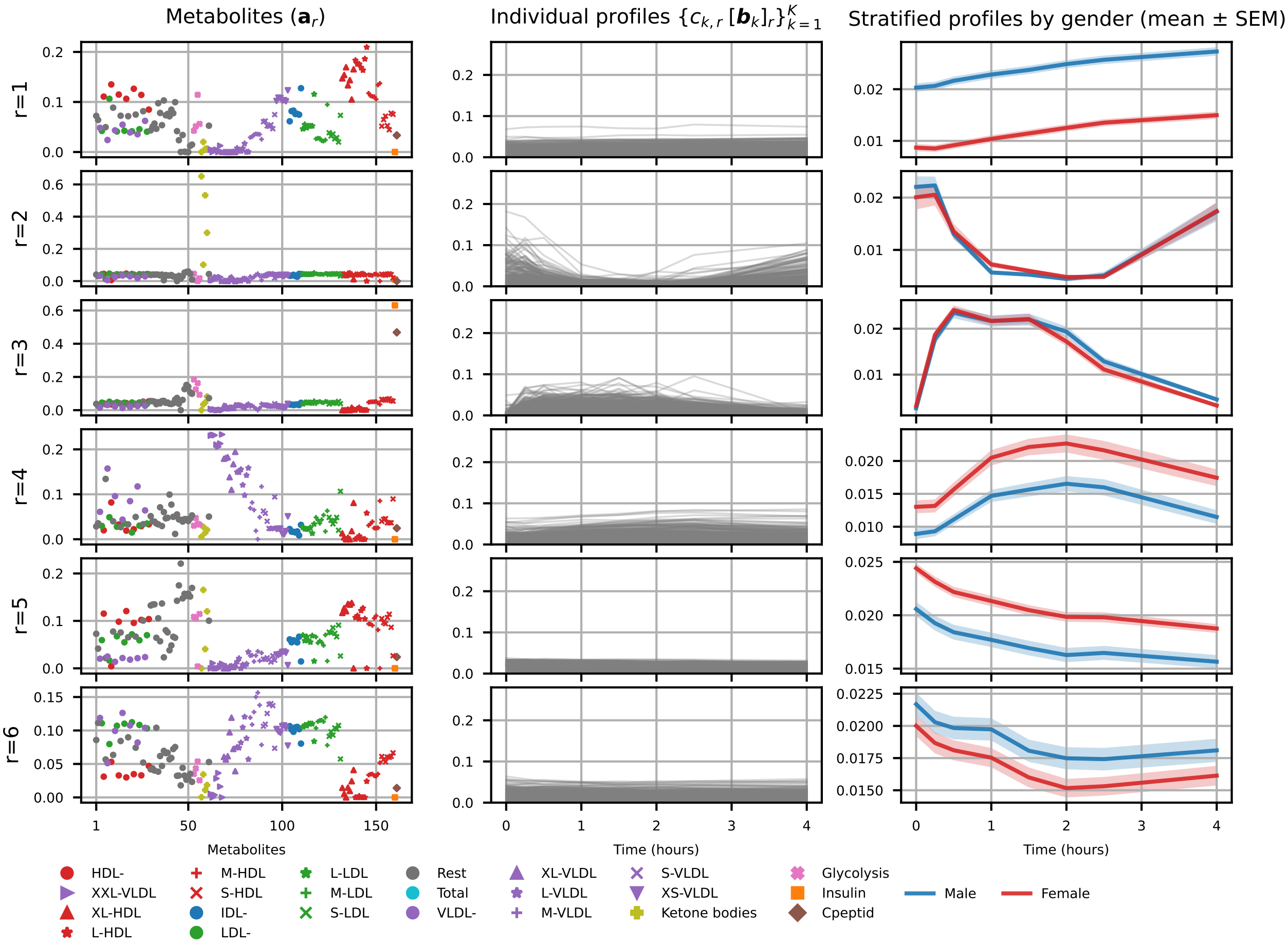

Figure S.11: Males and females. Components of a 6-component CMF model (with nonnegativity constraints in all modes). $\mathbf{a}_r$ denotes the pattern in the metabolites mode, where metabolites are colored by lipoprotein classes. Different shapes are used for lipoprotein subclasses. Subject-specific time profiles scaled by the corresponding subject scores, i.e., $c_{k,r}[\mathbf{b}_k]_r$ for each component are shown in the middle column. The last column shows scaled subject-specific time profiles colored according to gender.

2 Sensitization Data Analysis

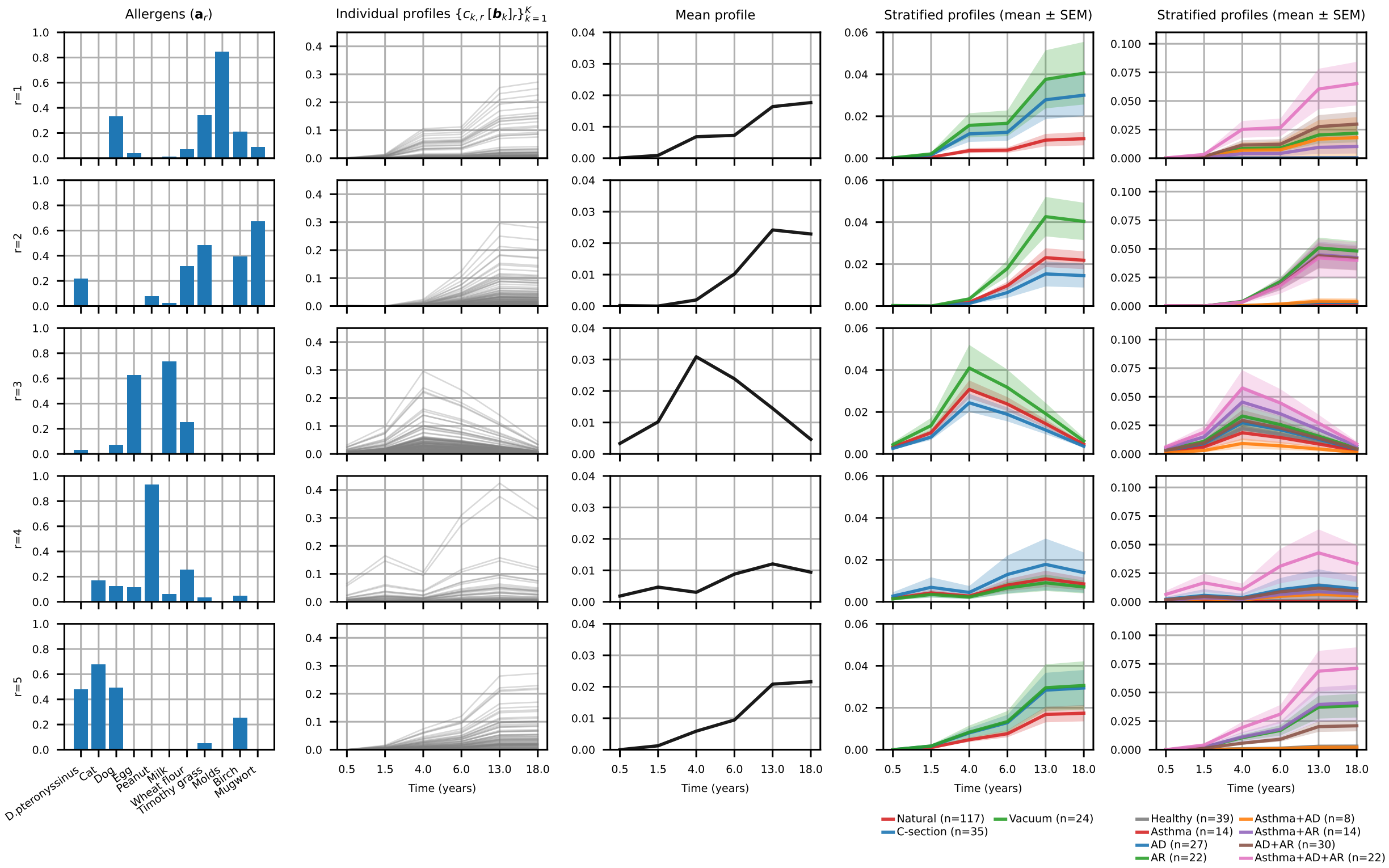

Figure S.12: Sensitization. Components of a 5-component CP model (with nonnegativity constraints in all modes) of the sensitization data. $\mathbf{a}_r$ denotes the pattern in the allergens mode. Subject-specific time profiles scaled by the corresponding subject scores, i.e., $c_{k,r}[\mathbf{b}_k]_r$ are shown in the middle column. Mean of scaled subject-specific profiles are plotted in the third column while the last two columns show mean (and standard error of mean) patterns of scaled subject-specific time profiles colored according to delivery/birth mode and disease groups.

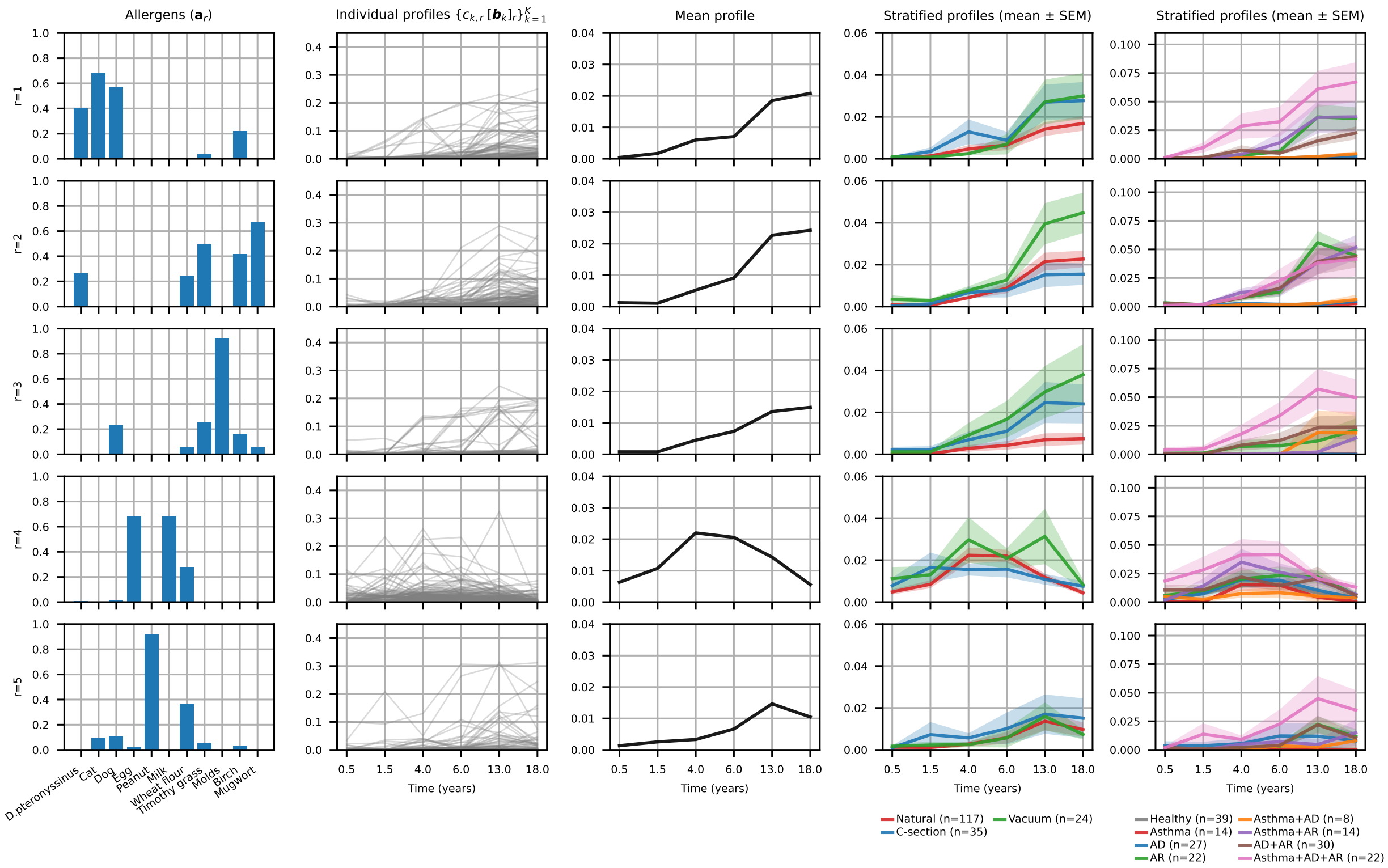

Figure S.13: Sensitization. Components of a 5-component PARAFAC2 model (with nonnegativity constraints in all modes). $\mathbf{a}_r$ denotes the pattern in the allergens mode. Subject-specific time profiles scaled by the corresponding subject scores, i.e., $c_{k,r}[\mathbf{b}_k]_r$ are shown in the middle column. Mean of scaled subject-specific profiles are plotted in the third column while the last two columns show mean (and standard error of mean) patterns of scaled subject-specific time profiles colored according to delivery/birth mode and disease groups.

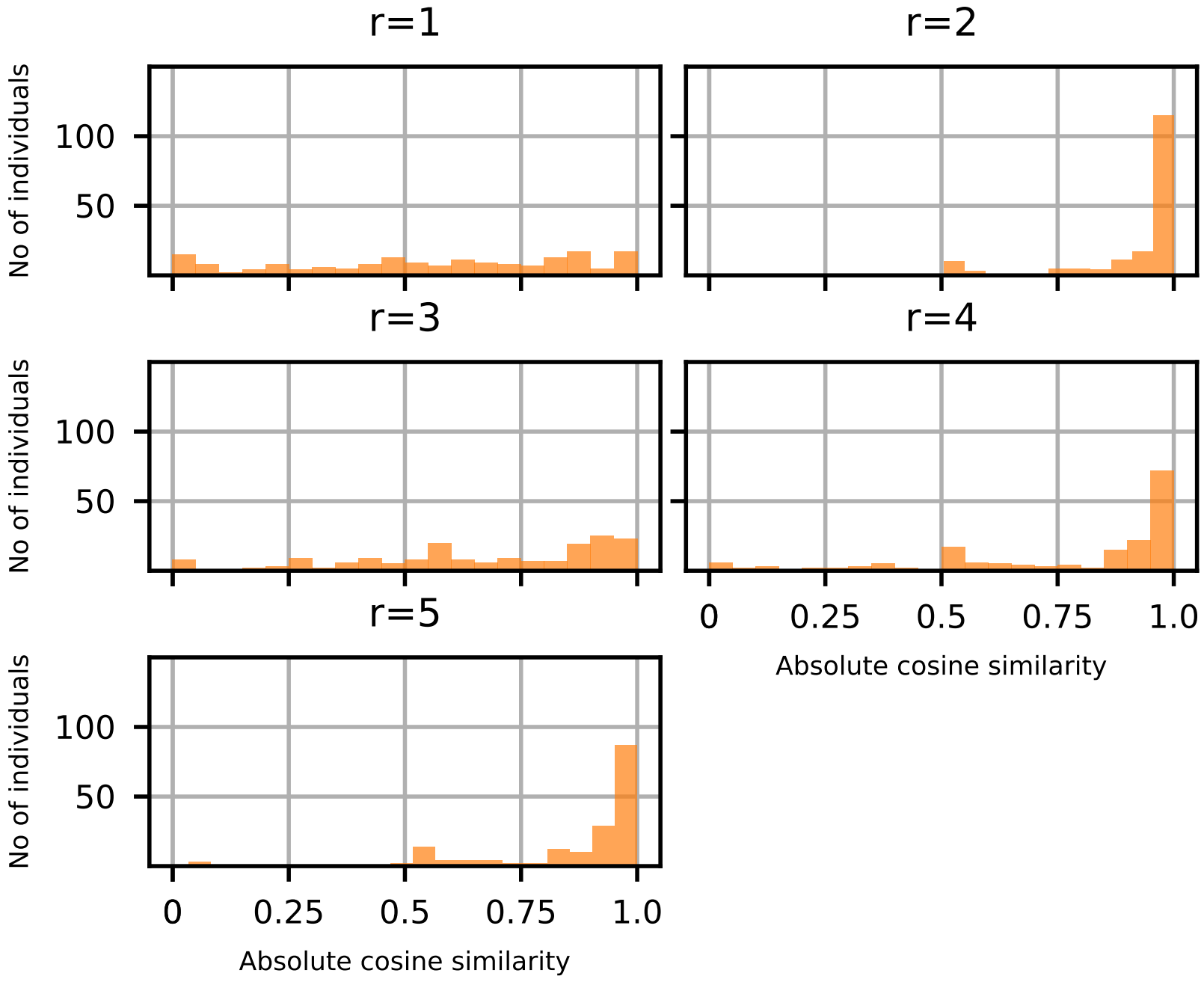

Figure S.14: Comparison of subject-specific time profiles ($c_{k,r}[\mathbf{b}_k]_r$) captured by CMF and PARAFAC2 for each component for sensitization, where each histogram contains 176 data points, one for each subject. The order is adjusted (following the order in the CMF model) so that matching components from different models are compared.

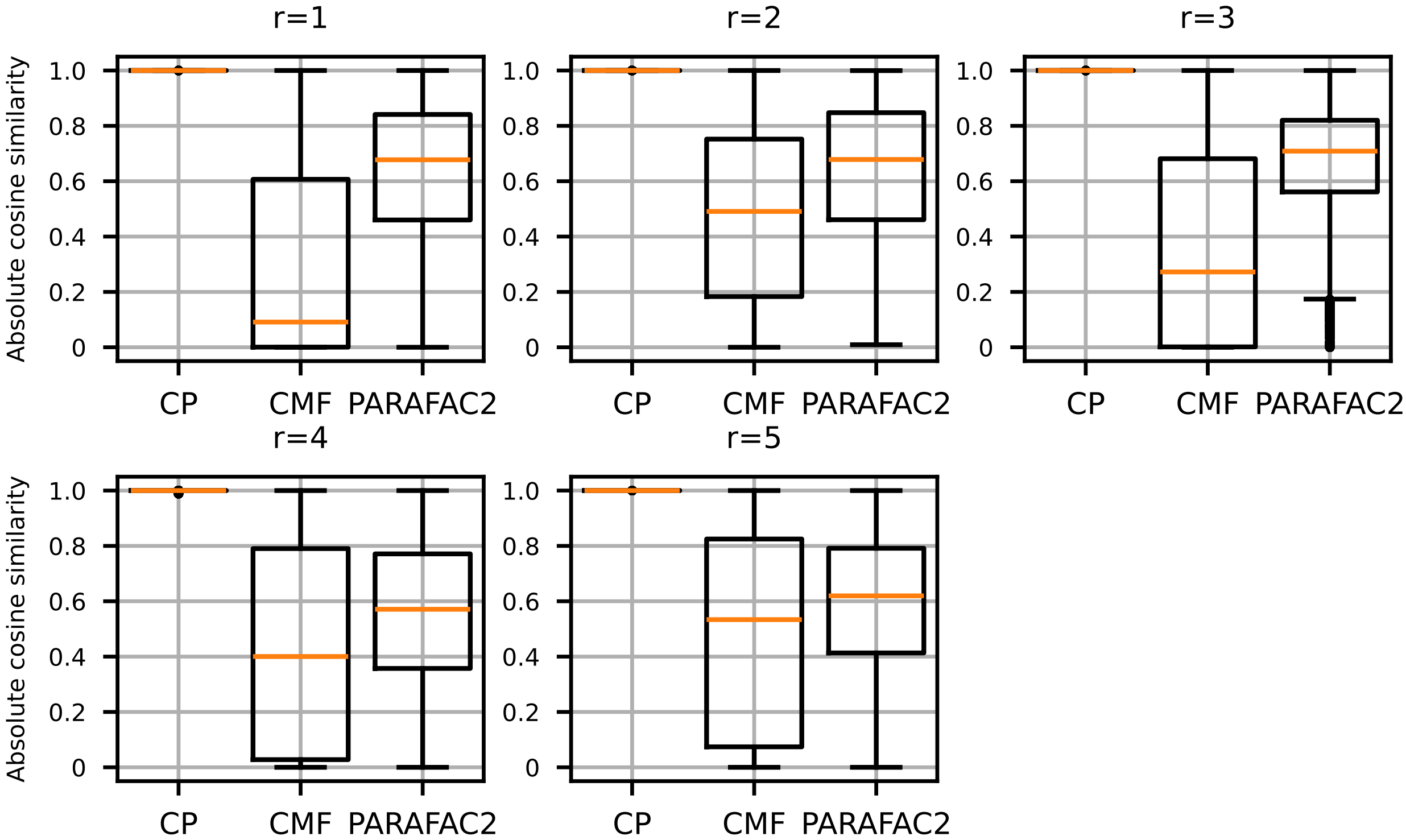

Figure S.15: Comparison of subject-specific time profiles extracted from the sensitization data within each component for each method in terms of absolute cosine similarity. The ordering of the components is determined by CMF, i.e. Fig. 6, and CP and PARAFAC2 are permuted so they match this exact order. CP does not allow structural variability, therefore the similarity between profiles is always one.

3 Synthetic dataset

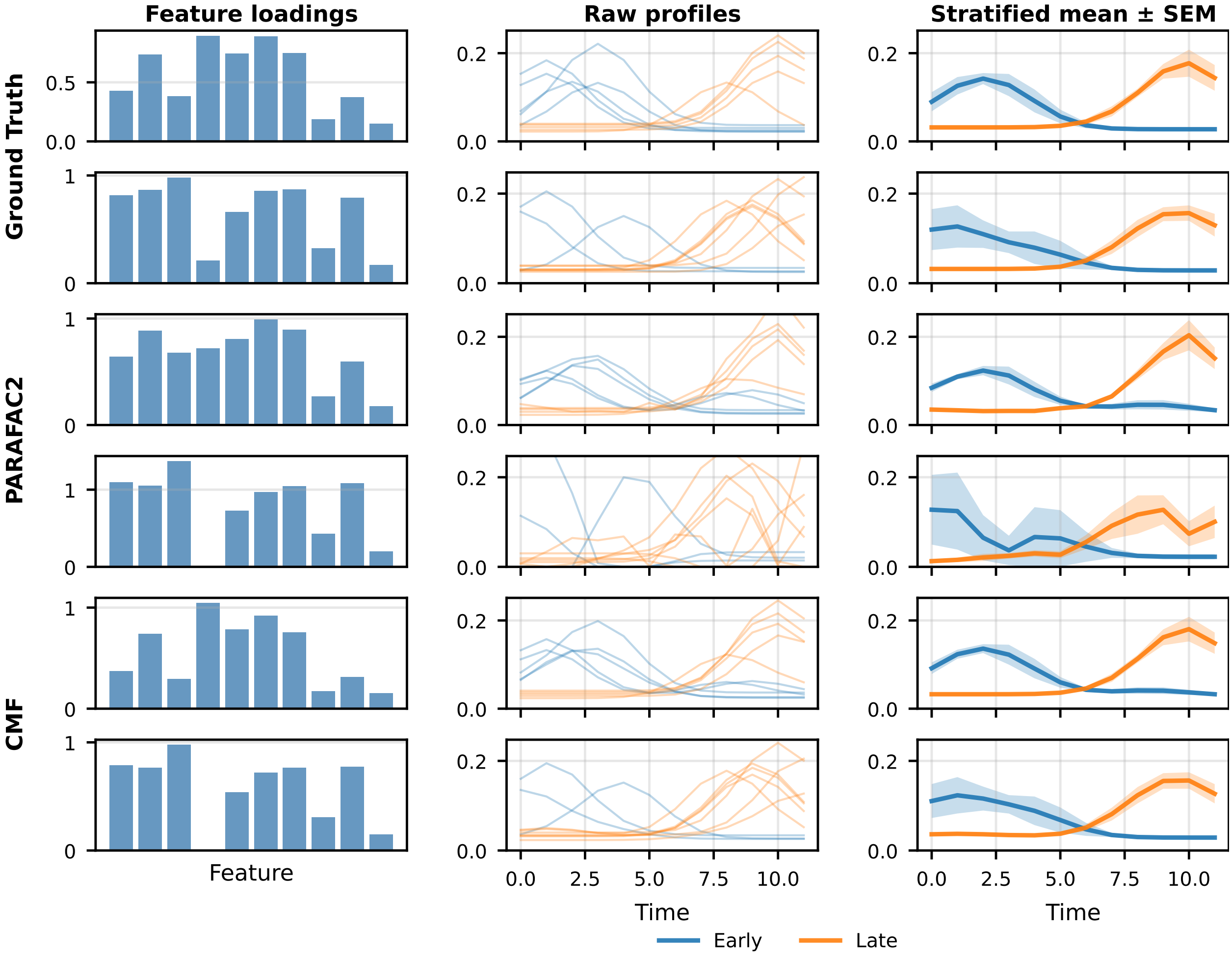

Figure S.16: Synthetic data experiment. The first two rows show the ground truth components of a synthetic dataset consisting of 10 features, 10 subjects, and 12 time points. In the first component, subjects are split 50/50 into early and late peakers; in the second, the split is 30/70. The full dataset is constructed by summing the two components. We then attempt to recover them using PARAFAC2 and CMF. Since the ground truth does not satisfy the PARAFAC2 constraint, PARAFAC2 recovers distorted profiles forced to conform to it, while CMF recovers the ground truth more accurately.
